# m6A regulator-mediated RNA methylation modification patterns are involved in immune microenvironment regulation of coronary heart disease

**DOI:** 10.3389/fcvm.2022.905737

**Published:** 2022-08-25

**Authors:** Zhaoshui Li, Yanjie Song, Meng Wang, Ruxin Shen, Kun Qin, Yu Zhang, Ting Jiang, Yifan Chi

**Affiliations:** ^1^Qingdao Medical College, Qingdao University, Qingdao, China; ^2^Heart Center Department, Qingdao Hiser Hospital Affiliated to Qingdao University, Qingdao, China

**Keywords:** m6A RNA methylation, immune microenvironment, LASSO regression, unsupervised clustering, coronary heart disease

## Abstract

**Background:**

Although the roles of m6A modification in the immune responses to human diseases have been increasingly revealed, their roles in immune microenvironment regulation in coronary heart disease (CHD) are poorly understood.

**Methods:**

The GSE20680 and GSE20681 datasets related to CHD were acquired from the Gene Expression Omnibus (GEO) database. A total of 30 m6A regulators were used to perform LASSO regression to identify the significant genes involved in CHD. Unsupervised clustering analysis was conducted using the m6A regulators to distinguish the m6A RNA methylation patterns in patients with CHD. The differentially expressed genes (DEGs) and biological characteristics, including GO and KEGG enrichment results, were assessed for the different m6A patterns to analyse the impacts of m6A regulators on CHD. Hub genes were identified, and subsequent microRNAs-mRNAs (miRNAs–mRNAs) and mRNAs-transcriptional factors (mRNA-TFs) interaction networks were constructed by the protein and protein interaction (PPI) network method using Cytoscape software. The infiltrating proportion of immune cells was assessed by ssGSEA and the CIBERSORT algorithm. Quantitative real-time PCR (qRT-PCR) was performed to detect the expression of the significant m6A regulators and hub genes.

**Results:**

Four of 30 m6A regulators (*HNRNPC, YTHDC2, YTHDF3*, and *ZC3H13*) were identified to be significant in the development of CHD. Two m6A RNA methylation clusters were distinguished by unsupervised clustering analysis based on the expression of the 30 m6A regulators. A total of 491 genes were identified as DEGs between the two clusters. A PPI network including 308 mRNAs corresponding to proteins was constructed, and 30 genes were identified as hub genes that were enriched in the bioprocesses of peptide cross-linking, keratinocyte differentiation. Twenty-seven hub genes were found to be related to miRNAs, and seven hub genes were found to be related to TFs. Moreover, among the 30 hub genes, eight genes were found to be upregulated in CHD, and three were found to be downregulated in CHD compared to the normal people. The high m6A modification pattern was associated with a higher infiltrated abundance of immune cells.

**Conclusion:**

Our findings demonstrated that m6A modification plays crucial roles in the diversity and complexity of the immune microenvironment in CHD.

## Introduction

Cardiovascular disease (CVD) is a chronic disease of the heart and circulatory system, and for many years, it has been the leading cause of premature death worldwide (1). Coronary heart disease (CHD) is the most common type of CVD (2), and it is caused by atherosclerotic plaque, atherosclerotic erosion, unstable atheroma, and vessel lumen stenosis (3). Arterial inflammation is caused by the recruitment of cells in the innate (e.g., monocytes, macrophages) and adaptive (e.g., CD4^+^ T helper cells and cytotoxic CD8^+^ T cells) immune systems into the intima of the artery (4). Therefore, understanding immune regulation in patients with CHD might be critical for revealing the molecular mechanism of its pathogenesis and might be conducive to uncovering novel immune therapies for CHD.

N6-methyladenosine (m6A) is the most prevalent type of internal RNA posttranscriptional modification in eukaryotic cells, and can occur in multiple types of RNA, including messenger RNA (mRNAs), ribosomal RNA (rRNAs), transfer RNA (tRNAs), long non-coding RNAs (lncRNAs) and microRNAs (miRNAs) (3, 5–7). This modification is a reversible process (8) that is controlled by three main components: adenosine methyltransferases (writers), m6A-binding proteins (readers), and m6A demethylating enzymes (erasers) (9, 10). The “writers” are m6A methyltransferases that catalyse the formation of the m6A modification (5), while the “erasers” are m6A demethylases that regulate the demethylation of the m6A modification (11, 12). The “readers” are m6A RNA binding proteins (RBPs) that recognize the target m6A-modified mRNA and promote its function in subsequent biological processes (13). Additionally, m6A RNA modification exerts diverse functions in normal bioprocesses and under disease conditions, including gene expression regulation, RNA stability, and RNA processing (14–17). Recently, m6A regulators have been reported to be linked to the immune microenvironment and participate in the regulation of human diseases (18). For instance, the results of Sun D's group suggested that m6A modification plays a key role in severe asthma, and may be able to guide future immunotherapy strategies (19).

Multiple independent studies have found increased levels of m6A RNA methylation in various CADs, including heart failure (20, 21), cardiac hypertrophy (22), myocardial infarction (23, 24), and CHD (25), indicating that m6A RNA methylation is closely linked to the occurrence and development of CADs. For example, the loss of the m6A methyltransferase METTL5 promotes cardiac hypertrophy through the epitranscriptomic control of SUZ12 expression (26). Zhao X's group found that METTL3 attenuated cardiomyocyte apoptosis in myocardial ischaemia–reperfusion (I/R) injury through miR-25-3p and miR-873-5p (27). Another study found that loss of m6A methyltransferase METTL3 promoted heart regeneration and repair after myocardial injury (28). Recently, a study by Mo's group (29) identified 304 CHD-associated m^6^A-SNPs that might alter the expression of local genes, which might subsequently affect CHD risk (29). Guo M's group found that the enhanced m6A RNA modification of circ_0029589 helped IFN regulatory factor-1(IRF-1) facilitate macrophage pyroptosis and inflammation in CHD (30). A study of the transcriptome-wide m6A landscape of CHD identified differentially methylated m6A sites in both mRNAs and lncRNAs between CHD and control groups (25). Another study found that loss of m6A methyltransferase METTL3 promoted heart regeneration and repair after myocardial injury (28). Although relevant studies have proposed the existence of expression changes in m6A regulators changes in CHD, their specific roles in the development and progression of CHD still need to be expounded.

This study aimed to systematically evaluate the modification patterns of m6A RNA methylation in patients with CHD by using bioinformatics to analyse CHD sequencing data from the Gene Expression Omnibus (GEO) database. The infiltration of immune cells in the different m6A patterns was further analyzed to illustrate the impacts of m6A RNA modification on the immune environment of CHD. The results lay a theoretical foundation for the regulation of m6A modification in the pathogenesis of CHD and provide a novel immune therapy approach for CHD.

## Materials and methods

### Data acquisition and preprocess

Coronary heart disease was retrieved from GEO database and human whole blood sequencing data were screened. The data used in this study were acquired from GSE159657 (31), GSE20680 (32), and GSE20681 (33) data sets of Gene Expression Omnibus (GEO) database (34). Among these, GSE20680 data set contains 143 human CHD samples and 52 control samples, GSE20686 data set contains 99 human CHD samples and 99 control samples, which were both sequenced *via* GPL4133 platform. The gene expression levels of the above two datasets were normalized signal intensity of Log2 transformation. R “sva” package (35) was used for the batch removal of the two data sets, and the integrated GEO data sets was obtained as the training set, that included 242 human CHD samples and 99 control samples. As a validation set, GSE159657 data set contains 8 human CHD samples and 10 control samples, with the sequencing platform of GPL24676. All data were obtained by R “GEOquery” (36) package. The detailed information of the GEO data sets was shown in Table 1.

**Table 1:** The detailed information of the GEO data sets.

**GEO ID**	**Platforms**	**Number of patients**	**Number of control**	**Species**
GSE20680	GPL4133	143	52	*Homo sapiens*
GSE20681	GPL4133	99	99	*Homo sapiens*
GSE159657	GPL24676	8	10	*Homo sapiens*

### Construction of forest model and nomogram model

The univariate logistic regression was used to select candidate m6A regulators from the 30 m6A regulators to predict the occurrence of CHD and the cut-off criteria are *P*-value < 0.05. The least absolute shrinkage and selection operator (LASSO) regression (37) was used for feature selection and dimension reduction to establish the predictive scoring formula. R “glmnet” package (38) was used to implement the method and select the best lambda value. After regression, only genes with non-zero coefficients were retained. Then, a rosette model was constructed based on the selected candidate m6A regulator to predict the prevalence of CHD by R “forestplot” package (39).

R “rms” package (40) was used for nomogram visualization to directly reflect the clinical significance of this model. Receiver operating characteristic (ROC) curve (41) was used to evaluate the distinguishing performance of the signature, and the area under the curve was calculated using R “pROC” package (42). Decision curve analysis (DAC) curve (43) was used to assess whether model-based decision making was beneficial to patients, drawing by R “ggDCA” package (44).

### Identification of m6A modification pattern

Unsupervised clustering analysis (45) was performed to identify diverse m6A modification pattern based on the expression of the 30 m6A regulators. The consistency clustering algorithm was used to evaluate the clustering number and robustness. R “Consensus Cluster Plus” package (46) was used to identify different m6A modification pattern. The above steps were performed 1,000 iterations to ensure the robustness of the classification. PCA was performed 20 times to further verify 30 m6A regulator expression patterns under different modification modes.

### Identification of DEGs between the two m6A modification patterns

To identify m6A regulator meditated genes, samples of two distinct m6A modification patterns were analyzed by the empirical Bayesian approach of the R “limma” package (47) to identify the differentially expressed genes (DEGs) between the two m6A modification patterns, with the threshold of log_2_FC > 1.5 and *P*_adj_ < 0.05. For the visualization of data, “Ggplot 2” R package was used to perform the volcano plot, and “Pheatmap” R package was used to perform the heatmap.

### Biological characteristics assessment between the two m6A modification patterns

The biological function of m6A phenotype-related genes was analyzed by Gene Ontology (GO) and Kyoto Encyclopedia of Genes and Genomes (KEGG) enrichment analysis. GO analysis is a common method for large-scale functional enrichment studies, including biological process (BP), molecular function (MF) and Cellular Component (CC) (48). KEGG is a widely used database that stores information about genomes, biological pathways, diseases and drugs (49). The gene name was firstly transited to be entrez ID using the R “org.hs.eg.db” package. R “clusterProfiler” package (50) was used for GO annotation analysis and KEGG pathway enrichment analysis of DEGs, FDR <0.05 was considered to be statistically significant.

To study the differences in biological processes between different m6A modification patterns, Gene Set Enrichment Analysis (GSEA) (51) was performed based on the data set of gene expression profiles in patients with CHD. The gene sets of “c2.cp.kegg.v7.4.symbols.gmt” and “c5.go.v7.2.symbols.gmt” were downloaded from MSigDB database (51) for the GSEA analysis, discovery rate <0.1, and *P*-value < 0.05 was considered to be statistically significant.

### Protein-protein interaction network construction

The STRING (https://cn.string-db.org/) (52) is a database that searches for known proteins and predicts interactions between proteins. STRING database was used, and genes with combined score >400 were selected to construct the protein-protein interaction (PPI) network. Cytoscape (v3.7.2) (53) was used for the visualization of PPI network. CytoHubba (54) is used to rank nodes according to their attributes in the network to identify the central elements in the network and discover the key targets and sub-networks of the complex network. The top 30 genes were identified ad hub genes.

For the miRNA-mRNA interaction network, miRNAs and lncRNAs were obtained from miRNet database (https://www.mirnet.ca/) (55), and the interaction network was performed by Cytoscape (v3.7.2). For the mRNA-transcription factor (mRNA-TF) interaction network, the relationship between TF and hub-genes was retrieved from miRNet database, the interaction network was performed by Cytoscape (v3.7.2). Different icons were used to label mRNAs, miRNAs and TFs.

### Functional similarity analysis

The functional similarity among proteins was evaluated using the geometric mean of semantic similarities in BPs, CCs, and MFs through GOSemSim R package (56).

### Immune cell infiltration analysis

Single sample gene set Enrichment analysis (ssGSEA) (57) is used to calculate the abundance of 28 immune cells in CHD with different m6A patterns. The immune reaction gene-sets were got from the ImmPort databses (http://www.immport.org) (58). The composition of immune cells in patients with different m6A patterns was visualized by box diagram. The difference in proportion of immune cells was calculated by Wilcoxon test, and *P*-value < 0.05 was considered as statistically significant.

CIBERSORT algorithm is a deconvolution algorithm for the expression matrix of immune cell subtypes based on the principle of linear support vector regression, using RNA-seq data to estimate the abundance of immune cells in samples (59). CIBERSORT algorithm was used to estimate the abundance of 22 types of immune cells in patients with different m6A patterns, and the composition of immune cells in patients with different m6A patterns was visualized by box diagram. The difference in proportion of immune cells was calculated by Wilcoxon test, and *P*-value < 0.05 was considered as statistically significant.

### The peripheral blood collection and the whole blood cells isolation

The peripheral blood was collected in the anticoagulant tube from 14 patients with CHD and 9 normal people. The whole blood cells were isolated form the collected peripheral blood using the Lymphocyte Separation Medium (#P8620, Solarbio) according to the manufacturer's protocol. The fresh blood and normal saline were mixed in a ratio of 1:1, the lymphocyte separation medium was carefully added to the top of the page, and centrifuged for 20 min, 1,500 r/min. The circular milky lymphocyte layer at the second layer was sucked out and placed in a new centrifuge tube containing 4–5 ml normal saline, and centrifuged for 20 min, 1,500 r/min. The desired whole blood cells were obtained after the precipitate was washed twice.

### Total RNA extraction, CDNA synthesis and quantitative real-time PCR

Total RNA was isolated using TRizol (#R401-01-AA, Vazyme Biotech) following the manufacturer's protocol. RNAs were then reverse transcribed using HiScript II Q RT SuperMix for qPCR Reverse Transcription Kit (#R223-01, Vazyme Biotech) following the manufacturer's protocol. Quantitative real-time PCR (qRT-PCR) was performed using AceQ® Universal SYBR® qPCR Master Mix (#Q511, Vazyme Biotech). Primers were synthesized by Integrated DNA Technologies. Target gene expression was normalized to the GAPDH reference gene and relative expression levels were determined using the 2^−ΔΔCt^ method. The primers used in qRT-PCR were listed in Supplementary Table 13.

### Statistical analysis

All data calculations and statistical analysis were performed using R programming (https://www.r-project.org/, version 4.1.1). For the comparison of the two groups of continuous variables, the statistical significance of the normally distributed variables was estimated using the independent Student *t* test, and the differences between the non-normally distributed variables were analyzed using the Mann-Whitney U test (i.e., Wilcoxon rank-sum test). The Chi-square test or Fisher's exact test was used to compare and analyze the statistical significance between the two groups of categorical variables. The correlation coefficients of different genes were calculated by Pearson correlation analysis. All statistical *P* values were bilateral, and *P* < 0.05 was considered statistically significant.

## Results

### Landscape of m6A RNA methylation regulators in CHD

**Figure 1:**
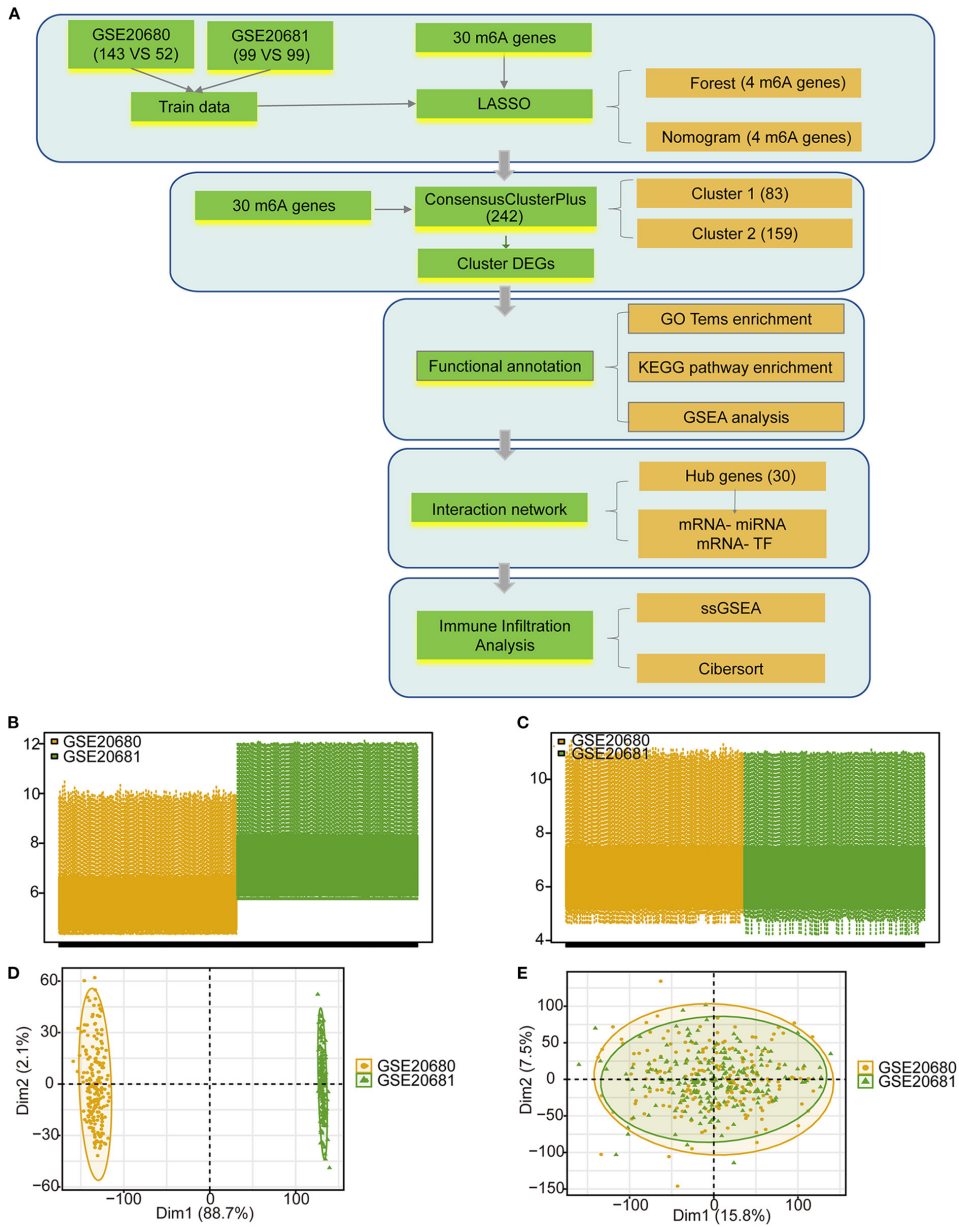
The data preprocessing and analysis process. **(A)** The work flow of this study. **(B)** Box plot showing the two GEO data sets before standardization. **(C)** Box plot showing the two GEO data sets after standardization. **(D)** PCA analysis of the two data sets before standardization. **(E)** PCA analysis of the two data sets after standardization.

The detailed processes used for the m6A regulator cluster construction and data analyses are shown in Figure 1A. The training data were obtained from the merged and batch-normalized GSE20680 and GSE20681 sets, and contained 242 disease samples and 151 normal samples (Figures 1B–E).

**Figure 2:**
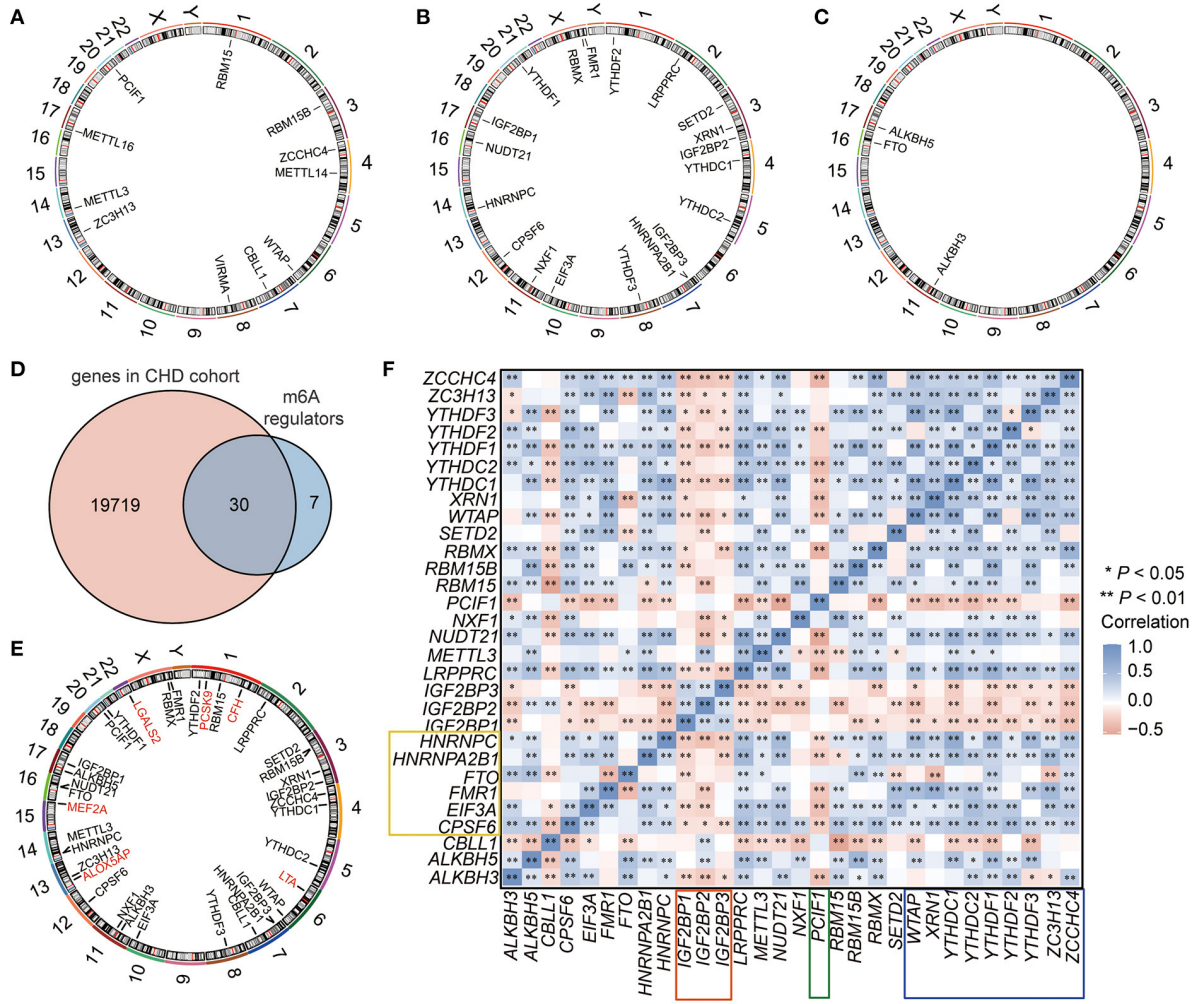
The landscape of m6A regulators in CHD. **(A–C)** The location of m6A RNA methylation writers **(A)**, readers **(B)**, and erasers **(C)** on the chromosome. **(D)** Venn showing the overlapped m6A regulators in the GEO-CHD cohort. **(E)** The location of m6A regulators and the CHD susceptibility genes. The red labeled genes were CHD susceptibility gene, and the black labeled genes were m6A regulators. **(F)** The correlation of the m6A regulators in CHD.

A total of 37 m6A regulators (60) were extracted from the dataset, including 11 writers (*METTL3, METTL14, WTAP, VIRMA, ZC3H13, CBLL1, RBM15, RBM15B, METTL16, ZCCHC4*, and *PCIF1*) (Figure 2A; Supplementary Table 1), 23 readers (*YTHDF2, FMR1, RBMX, YTHDF1, IGF2BP1, HNRNPC, NUDT21, CPSF6, NXF1, EIF3A, YTHDF3, HNRNPA2B1, IGF2BP3, YTHDC2, YTHDC1, IGF2BP2, XRN1, SETD2, LRPPRC, PRRC2, SRSF3, TRMT112*, and *SRSF10*) (Figure 2B; Supplementary Table 1, among these, only 19 genes can be annotated for location on human chromosome), and 3 erasers (*FTO, ALKBH5*, and *ALKBH3*) (Figure 2C; Supplementary Table 1). However, 30 m6A regulators were overlapped with genes in the CHD expression profiles, and these m6A regulators were used for the subsequent analysis (Figure 2D; Supplementary Table 2). Seven CHD susceptibility genes (*ALOX5AP, LTA4, MEF2A, LTA, LGALS2, PCSK9, CFH*) (61) were used to analyse the possible susceptibility to CHD associated with these m6A regulators by mapping the chromosomal locations of these genes and the m6A genes (Figure 2E). Among the examined genes, *PCSK9* was obviously close to the m6A “reader” *YTHDF2*, with both located on chr1. *ALOX5AP* and the “writer” *ZC3H13* were both located on chr13. Other CHD susceptibility genes were located at varying close distances to m6A regulators (Figure 2E). These results suggested that the m6A regulators may have potential regulatory effects on these genes. Then, we analyzed the correlations of these m6A regulators in the 242 CHD samples, and the results showed that these m6A regulators were strongly correlated in patients with CHD (Figure 2F). Among the examined genes, 3 m6A RNA methylation writers (*WTAP, ZCCHC4*, and *ZCCHC4*) and 6 m6A RNA methylation readers (*XRN1*,*YTHDF1, YTHDF2, YTHDF3, YTHDC1*, and *YTHDC2*) were significantly and positively correlated with each other (marked by blue line, Figure 2F); the writer *PCIF1* was negatively correlated with almost all regulators (marked by green line, Figure 2F); the readers *IGF2BP1-3* had similar patterns that were negatively correlated with other regulators (marked by red line, Figure 2F); while *HNRNPC, CPSF6, EIF3A, HNRNPA2B1*, and *FMR1* had similar patterns that were also positively correlated with other regulators (marked by yellow line, Figure 2F). The results indicated that these similar m6A RNA methylation modifiers might have a synergistic effect on gene expression regulation by m6A RNA methylation.

### m6A RNA methylation regulators are involved in CHD progression

**Figure 3:**
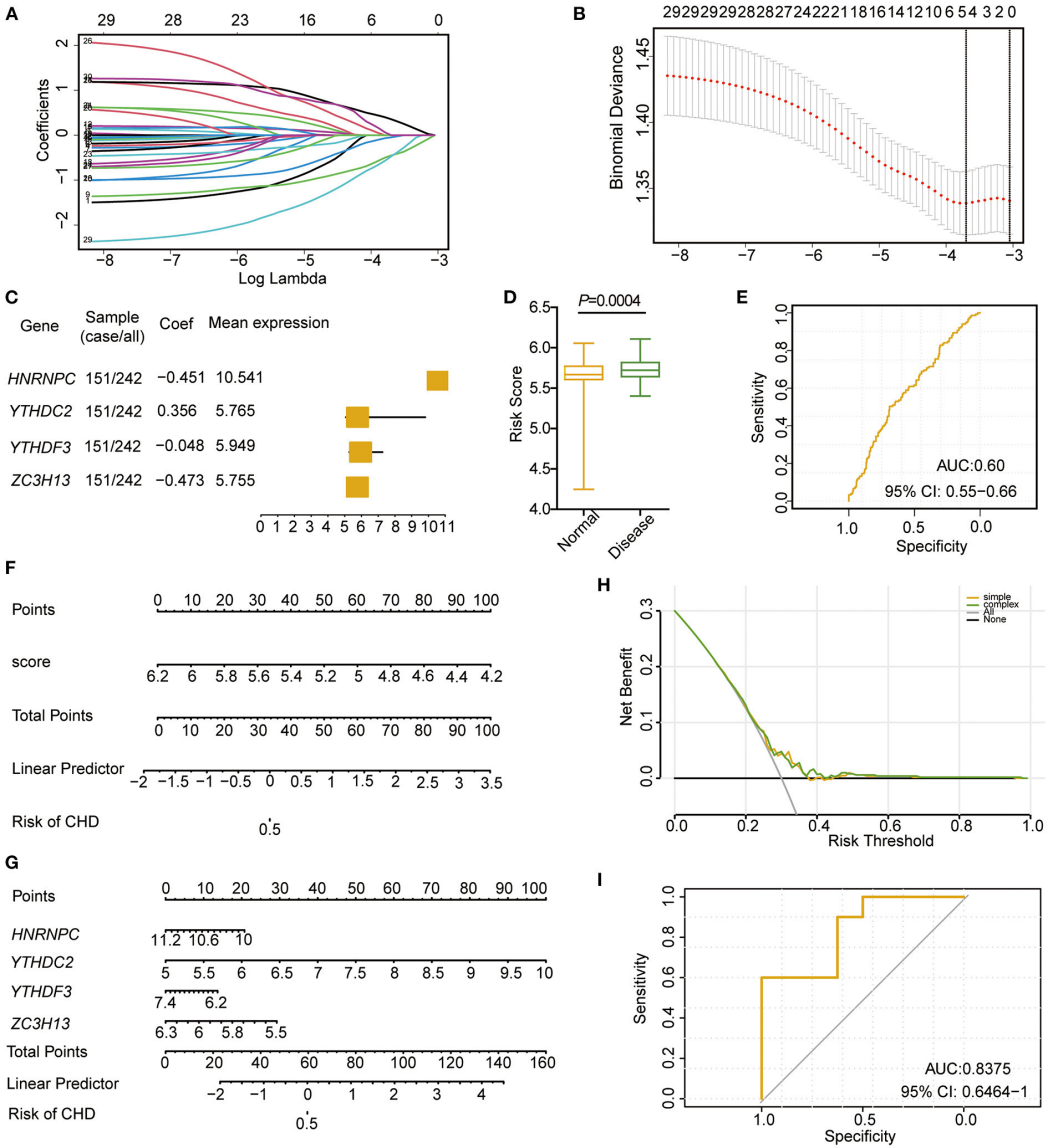
Cox model construction based on m6A regulators in CHD. **(A,B)** LASSO regression to identify significant m6A RNA methylation modification genes related to CHD. **(C)** Forest diagram showing the four significant m6A regulators in patients with CHD. **(D)** The risk score between the disease and normal samples. **(E)** ROC curve of the four significances. **(F)** Nomogram of diagnostic scores for patients with CHD. **(G)** Nomogram of the four m6A regulator significance for the diagnosis of CHD. **(H)** PCA analysis of the nomogram model. **(I)** ROC curve of the cox model of the validation set (GSE159657).

To analyse the roles of these m6A regulators in CHD, least absolute shrinkage and selection operator (LASSO) regression was performed on the 30 m6A regulators for feature selection and dimension reduction to identify the significant genes, and we found that four genes, including *HNRNPC, YTHDC2, YTHDF3*, and *ZC3H13*, were significant in CHD (Figures 3A–C; Supplementary Table 3). The classifier consisted of these four significant genes that could well distinguish healthy people from CHD patients, with CHD patients presenting higher risk scores than healthy people (Figure 3D). The receiver operating characteristic (ROC) curve also illustrated that the model could better predict patients with CHD (Figure 3E). The nomogram line diagram model was applied to predict the occurrence of CHD (Figures 3F,G), and it showed that four significant m6A-related genes had predictive value for CHD. The decision curve analysis (DCA) analysis also verified that decisions based on the nomogram model may be beneficial for CHD patients (Figure 3H). Finally, the risk model based on the four m6A-related significant genes was validated based on the GSE159657 dataset, and the ROC curve suggested the feasibility of the model (Figure 3I). These results indicate that m6A RNA modification plays important roles in the development and progression of CHD.

### m6A RNA modification clusters in CHD

**Figure 4:**
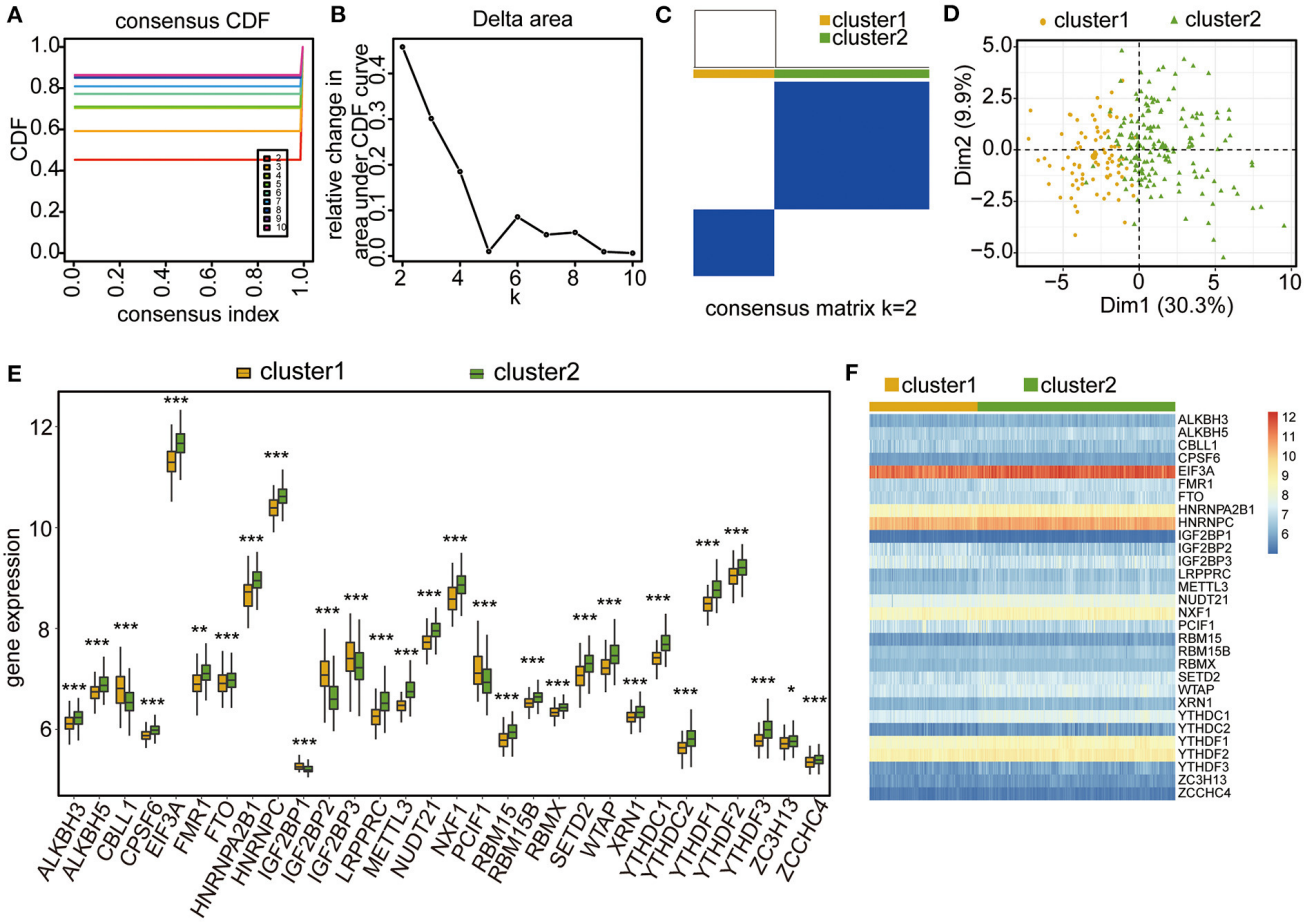
Unsupervised clustering analysis of CHD samples based on the four-gene significance. **(A–C)** Unsupervised clustering result of CHD samples. **(D)** PCA analysis of the two m6A RNA modification patterns. **(E)** Box plot showing the expressional levels of the 30 m6A regulators in the two m6A RNA modification patterns. The statistical significance was calculated *via* Wilcoxon rank sum test, ***P < 0.001, **P < 0.01, *P < 0.05. **(F)** Heatmap showing the expressional levels of the 30 m6A regulators in the two m6A RNA modification patterns.

Based on the expression levels of the 30 m6A regulators, unsupervised consensus clustering was conducted to investigate the m6A RNA modification clusters in the CHD samples. Two distinct subgroups were identified based on qualitative differences in expression among the 30 m6A-related genes, with 83 samples included in Cluster 1 and 159 samples included in Cluster 2 (Figures 4A–C; Supplementary Table 4). The principal component analysis (PCA) results showed that the two clusters had visible distinctions (Figure 4D). All 30 m6A regulators had remarkable differences in expression between the two m6A modification clusters (Figures 4E,F), and the four significant m6A-related genes were expressed at higher levels in Cluster 2 than in Cluster 1 (Figure 4E). These results validate the different m6A RNA modification patterns in the two clusters of CHD patients.

### Biological enrichment analysis of distinct m6A RNA modification clusters

**Figure 5:**
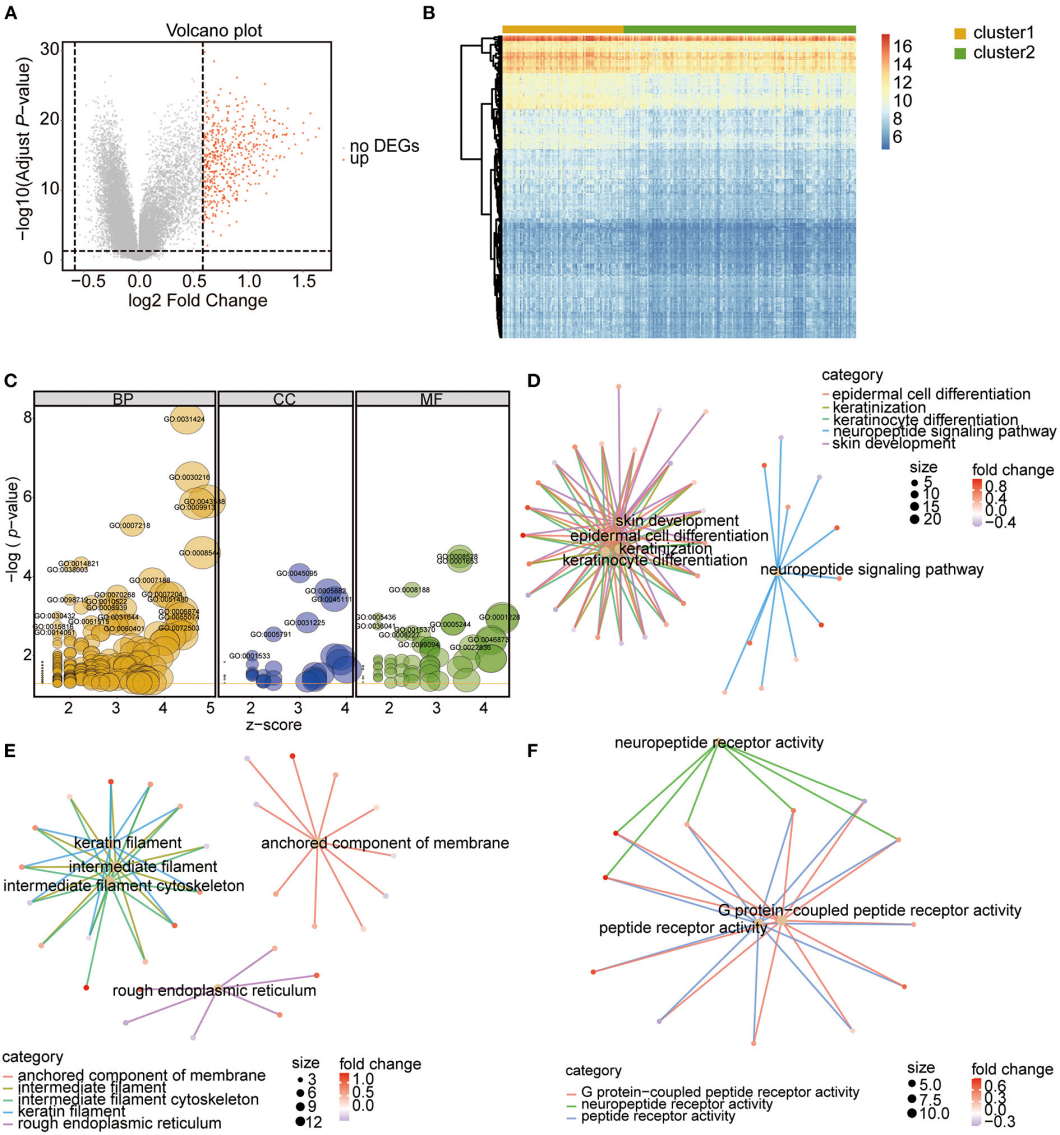
Identification and function analysis of DEGs between the two m6A RNA modification patterns. **(A)** Volcano Plot showing the DEGs in cluster 1 vs. cluster 2 subgroups. The red dots represent up-regulated genes, the gray dots represent genes with no significant difference. DEGs were identified with the threshold of adjust *P*-value < 0.05 and Fold change > 1.5. **(B)** Heatmap showing the DEGs in cluster 1 vs. cluster 2 subgroups. **(C)** GO functional enrichment analysis of the DEGs. The node size represents the number of genes contained in the current GO Term. BP, biological process; CC, cellular component; MF, molecular function. **(D–F)** Top 5 terms of BP **(D)**, CC **(E)**, and MF **(F)**.

To assess the biological characteristics of the two m6A RNA modification clusters, the differentially expressed genes (DEGs) between the clusters were identified. Based on the threshold of a fold change >1.5 and an adjusted *P* < 0.05, a total of 491 genes were found to be significantly differentially expressed between the two clusters (Figures 5A,B; Supplementary Table 5). The subsequent Gene Ontology (GO) enrichment results showed that these DEGs belonged to the biological processes of epidermal cell differentiation, keratinocyte differentiation, keratinization, and skin development signaling pathways (Figures 5C,D; Table 2; Supplementary Table 6). Additionally, these DEGs were enriched in the cellular component categories of anchored components of the membrane, intermediate filaments, intermediate cytoskeleton filaments, keratin filaments, and rough endoplasmic reticulum (Figures 5C,E; Table 2; Supplementary Table 6), and the molecular function categories of G-protein coupled receptor activity, neuropeptide receptor activity, and peptide receptor activity (Figures 5C,F; Table 2; Supplementary Table 6).

**Table 2:** Top 10 GO and KEGG enrichment of DEGs.

**Category**	**ID**	**Description**	***p*-value**
BP	GO:0031424	Keratinization	1.04E-08
BP	GO:0030216	Keratinocyte differentiation	3.07E-07
BP	GO:0043588	Skin development	1.26E-06
BP	GO:0009913	Epidermal cell differentiation	1.43E-06
BP	GO:0007218	Neuropeptide signaling pathway	5.07E-06
BP	GO:0008544	Epidermis development	2.49E-05
BP	GO:0014821	Phasic smooth muscle contraction	4.64E-05
BP	GO:0038003	Opioid receptor signaling pathway	5.42E-05
BP	GO:0007188	Adenylate cyclase-modulating G protein-coupled receptor signaling pathway	0.000123
BP	GO:0007204	Positive regulation of cytosolic calcium ion concentration	0.00027
BP	GO:0031424	Keratinization	1.04E-08
BP	GO:0030216	Keratinocyte differentiation	3.07E-07
BP	GO:0043588	Skin development	1.26E-06
CC	GO:0045095	Keratin filament	8.18E-05
CC	GO:0005882	Intermediate filament	0.000227
CC	GO:0045111	Intermediate filament cytoskeleton	0.000370
CC	GO:0031225	Anchored component of membrane	0.001451
CC	GO:0005791	Rough endoplasmic reticulum	0.002869
CC	GO:0001533	Cornified envelope	0.009983
CC	GO:0097060	Synaptic membrane	0.010234
CC	GO:0005667	Transcription regulator complex	0.010977
CC	GO:0017146	NMDA selective glutamate receptor complex	0.014357
CC	GO:0062023	Collagen-containing extracellular matrix	0.014529
MF	GO:0008528	G protein-coupled peptide receptor activity	3.09E-05
MF	GO:0001653	Peptide receptor activity	4.02E-05
MF	GO:0008188	Neuropeptide receptor activity	0.000214
MF	GO:0005436	Sodium:phosphate symporter activity	0.001049
MF	GO:0001228	DNA-binding transcription activator activity, RNA polymerase II-specific	0.001051
MF	GO:0001216	DNA-binding transcription activator activity	0.001167
MF	GO:0005244	Voltage-gated ion channel activity	0.001600
MF	GO:0022832	Voltage-gated channel activity	0.001600
MF	GO:0036041	Long-chain fatty acid binding	0.001766
MF	GO:0015370	Solute: sodium symporter activity	0.002657

**Figure 6:**
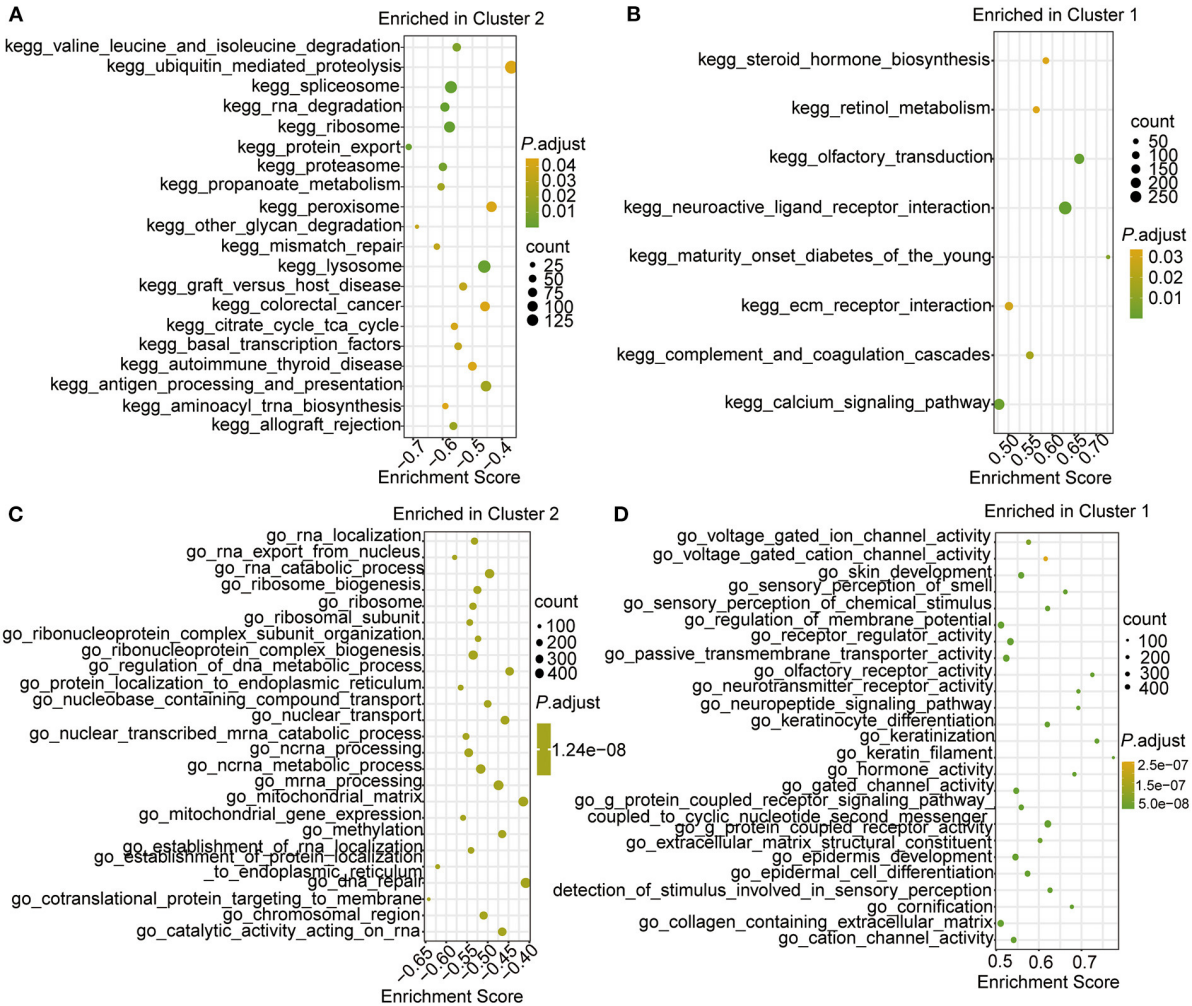
The GSEA enrichment analysis in the two clusters. **(A,B)** Top 10 GSEA-KEGG enrichment of genes in cluster 2 **(A)** and cluster 1 **(B)**. **(C,D)** Top 10 GSEA-GO enrichment of genes in cluster 2 **(C)** and cluster 1 **(D)**.

Then, gene set enrichment analysis (GSEA) enrichment was performed for the two clusters, and the results showed that the Kyoto Encyclopedia of Genes and Genomes (KEGG) pathways of ubiquitin-mediated proteolysis, peroxisomes, colorectal cancer, and autoimmune thyroid disease were enriched in Cluster 2 (Figure 6A; Supplementary Table 7), whereas the pathways of steroid hormone biosynthesis and retinol metabolism were enriched in Cluster 1 (Figure 6B; Supplementary Table 7). In the biological processes category, the terms DNA repair, mitochondrial matrix, and RNA catabolic process were enriched in Cluster 2 (Figure 6C; Supplementary Table 8), whereas the terms voltage-gated cation channel activity and skin development were enriched in Cluster C1 (Figure 6D; Supplementary Table 8). These results indicate that the examined m6A RNA modifications influenced these biological processes and pathways, which further impacted the development and progression of CHD.

### Hub gene identification and MiRNA–MRNA and MRNAs–transcription factor network construction

**Figure 7:**
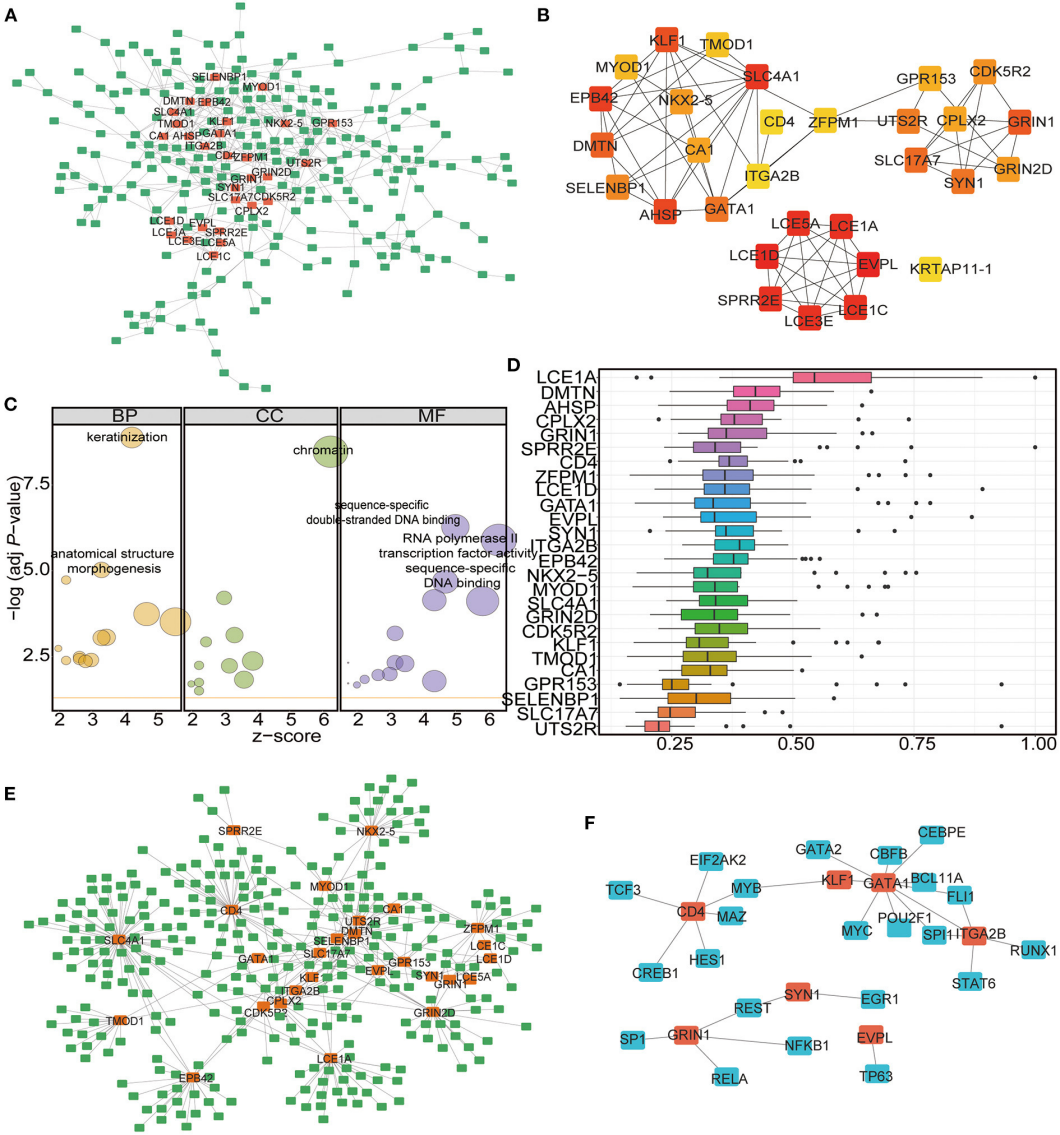
Identification of the hub-genes and the miRNA-mRNA-TF network construction. **(A)** PPI network analysis of DEGs in the two clusters. **(B)** Hub-gene network among the DEGs. **(C)** GO functional enrichment analysis of the Hub-genes. The node size represents the number of genes contained in the current GO Term. BP: biological process; CC: cellular component; MF: molecular function. **(D)** Functional similarity analysis of the hub-genes. The horizontal axis is the correlation size, the vertical axis is the gene name. **(E)** The miRNA-mRNA interaction network of the hub-genes. The green label represents miRNAs, the orange label represents mRNAs. **(F)** The mRNA-TF interaction network of the hub-genes. The blue label represents TFs, the orange label represents mRNAs.

To identify the hub genes among the DEGs, a protein–protein interaction (PPI) network was constructed by using the STRING database and Cytoscape software. A total of 499 interaction pairs and 308 genes were identified in the PPI network (Figure 7A). Among these genes and interactions, NK2 homeobox 5 (*NKX2-5*) was closely related to 16 DEGs while glutamate ionotropic receptor NMDA type subunit 1 (*GRIN1*), solute carrier family 4 member 1 (*SLC4A1*), and urotensin 2 receptor (*UTS2R*) were closely related to 15 DEGs (Figure 7A). CytoHubba was used to extract the functional interaction subnet containing 30 hub genes (Figure 7B; Supplementary Table 9). The GO enrichment results showed that these hub genes were linked to the biological processes of keratinization, chromatin, and sequence-specific double-stranded DNA binding (Figure 7C; Table 3; Supplementary Table 10). Moreover, we ranked the hub genes based on the average functional similarity (Figure 7D). Late cornified envelope 1A (*LCE1A*) was the top protein that potentially played a key role in CHD (Figure 7D). Moreover, the ROC results showed that these hub genes could distinguish the two RNA modification patterns well (Supplementary Figure 1).

**Table 3:** Top 3 GO enrichment of hub-genes.

**Category**	**ID**	**Description**	***p*-value**
BP	GO:0031424	Keratinization	1.31E-09
BP	GO:0009653	Anatomical structure morphogenesis	9.44E-06
CC	GO:0000785	Chromatin	3.40E-09
MF	GO:1990837	Sequence-specific double-stranded DNA binding	5.26E-07
MF	GO:0000981	RNA polymerase II transcription factor activity, sequence-specific DNA binding	1.27E-06

The important roles of microRNAs (miRNAs) in the development and treatment of human diseases have been determined in recent years. A previous study predicted potential miRNA-disease associations by using a novel unsupervised deep learning framework with a variational autoencoder (62). To study the ncRNAs associated with the selected hub genes, we acquired the associated miRNAs and lncRNAs from the miRNet database and constructed the mRNA-miRNA-lncRNA interaction network (Supplementary Figure 2). Among the hub genes, 27 were found to be correlated with ncRNAs. A total of 308 miRNAs (Supplementary Table 11 sheet 1) and 883 lncRNAs (Supplementary Table 11 sheet 1) were found to be associated with the 27 mRNAs (Supplementary Figure 2). We then analyzed the miRNA–mRNA interaction network, which included 396 interactions involving 27 mRNAs and 308 miRNAs (Figure 7E; Supplementary Table 11 sheet 2). According to the results, *SLC4A1* had the largest number of interactional miRNAs at 63, while hsa-mir-146a-5p had the most target mRNAs at 8 (Figure 7E).

Then, we established the mRNA–TF network to analyse the regulation of gene expression modes by the hub genes. The mRNA–TF interaction network included 29 interactions involving 7 mRNAs and 23 TFs (Figure 7F; Supplementary Table 12). According to the results, GATA binding protein 1 (*GATA1*) regulated 9 TFs, and it also served as a TF and interacted with 3 hub genes (Figure 7F). The results suggest that these m6A-related hub genes are involved in the gene regulatory network in CHD.

### Immune microenvironment characteristics in the two m6A RNA modification clusters

**Figure 8:**
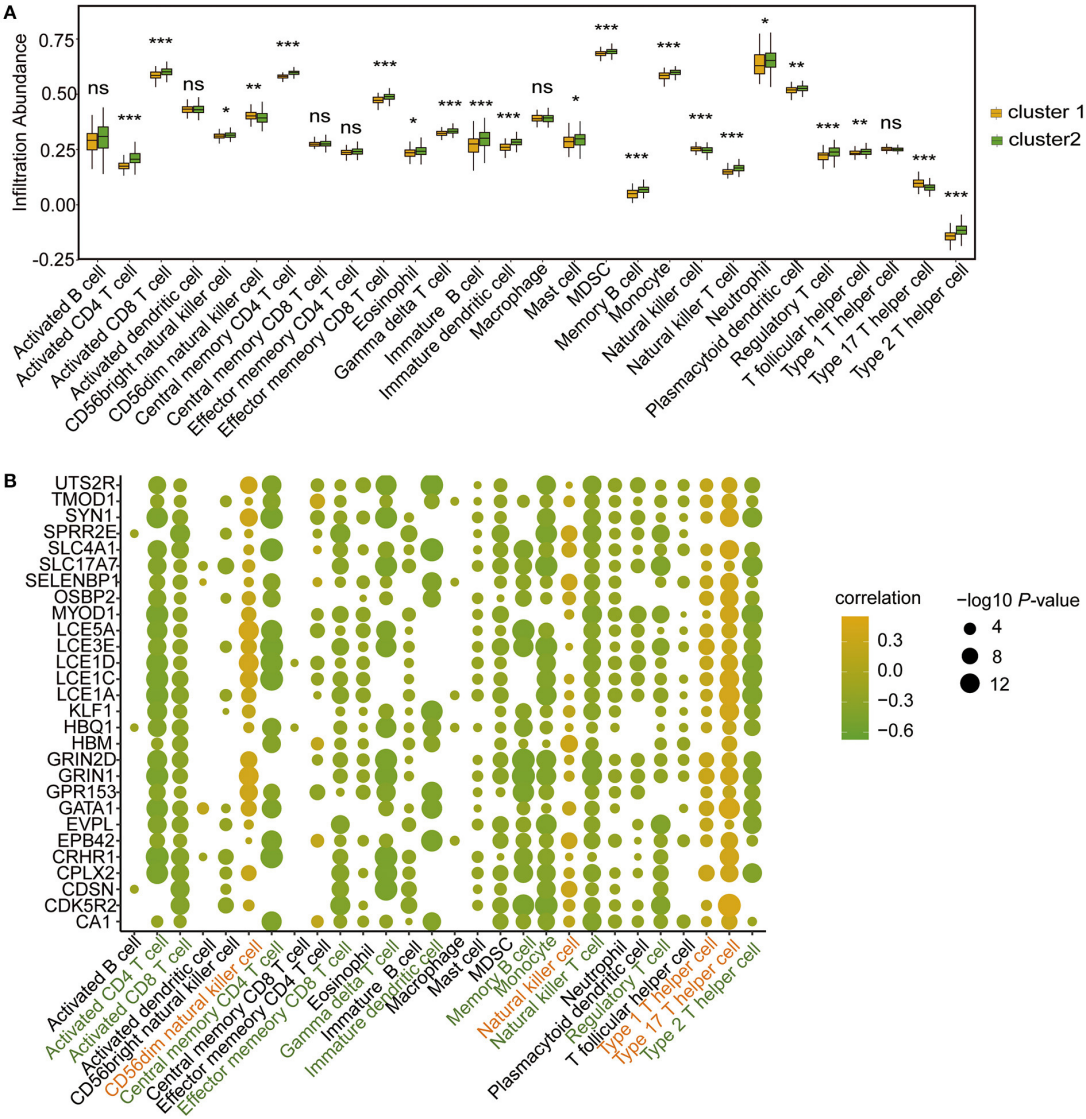
Immune microenvironment characteristics of immune cells in CHD patients of the two m6A RNA modification patterns according to ssGSEA. **(A)** Box plot showing the immune cell levels in the two m6A RNA modification patterns based on ssGSEA result. The statistical significance was calculated *via* Wilcoxon rank sum test, ***P < 0.001, **P < 0.01, *P < 0.05. **(B)** The correlation between hub-genes and immune cell levels. The horizontal axis represents immune cells, the vertical axis represents Hub gene, the node color represents correlation, and the node size represents significance level.

To further determine the differences in immune microenvironment characteristics between the two m6A RNA modification clusters, single-sample GSEA (ssGSEA) was performed to calculate the infiltrated proportions of 28 types of immune cells. The results showed that the infiltrated proportions of multiple types of immune cells were significantly lower in Cluster 1 than in Cluster 2 (Figure 8A). Moreover, the hub genes were strongly correlated with the immune cell infiltration abundance (Figure 8B). For example, the infiltrated abundance of activated CD4 T cells, activated CD8 T cells, effector memory CD8 T cells, central memory CD4 T cells, regulatory T cells, gamma delta T cells, immature dendritic cells, memory B cells, monocytes, natural killer T cells, and type 2 T helper cells were significantly and negatively correlated with the hub genes (Figure 8B, green). The abundances of infiltrated natural killer T cells, CD56dim natural killer cells, type 1 T helper cells and type 17 T helper cells were positively correlated with the hub genes (Figure 8B, red).

**Figure 9:**
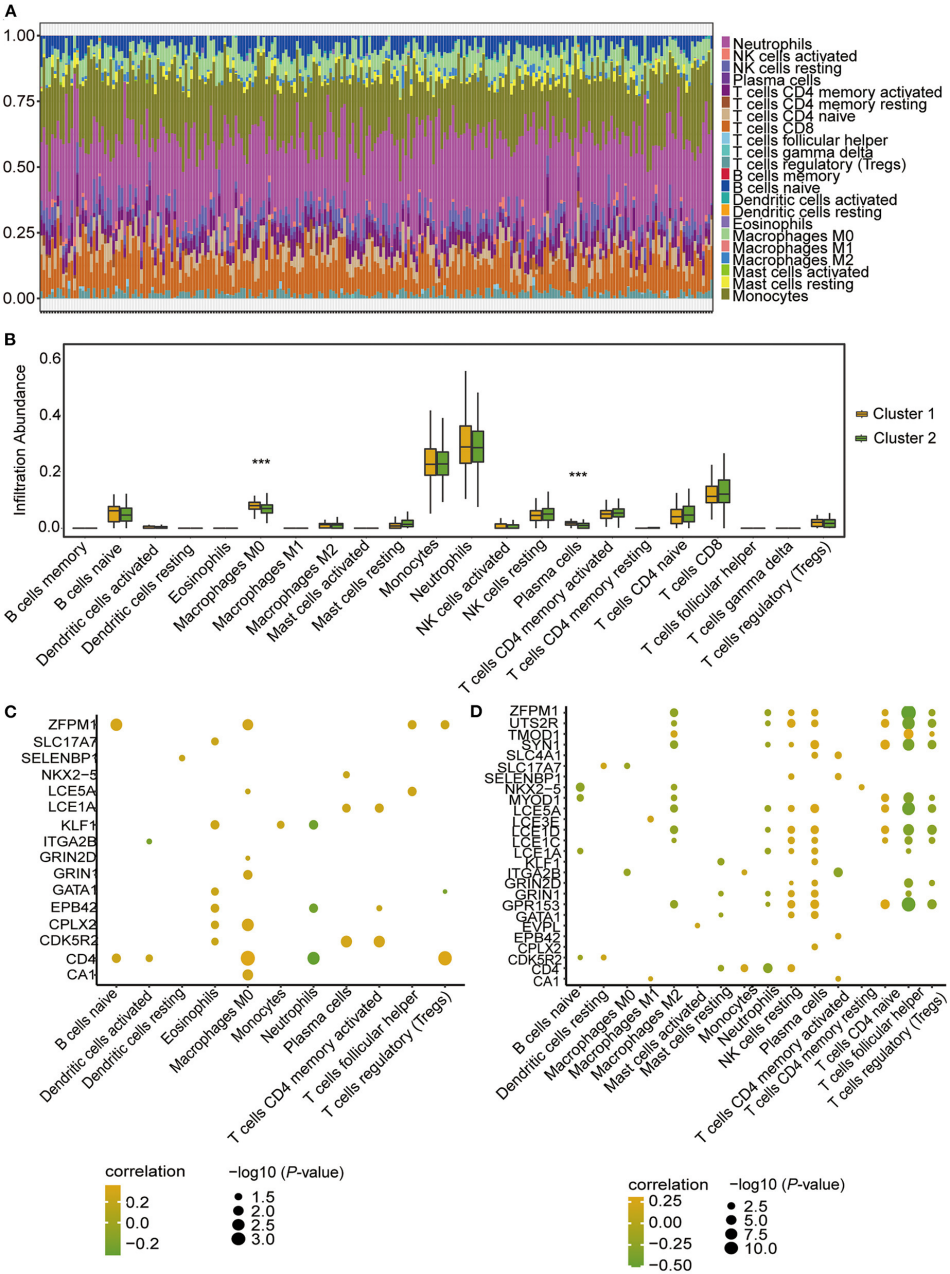
Infiltrating proportion of immune cells in CHD patients of the two m6A RNA modification patterns according to CIBERSORT algorithm. **(A)** The landscape of immune cells infiltration in patients with CHD based on CIBERSORT algorithm. **(B)** Box plot showing the proportion of immune cells infiltration between cluster 1 and cluster 2. The statistical significance was calculated *via* Wilcoxon rank sum test, ***P < 0.001. **(C,D)** The correlation between hub-genes and immune cell levels in cluster 1 **(C)** and cluster 2 **(D)**. The horizontal axis represents immune cells, the vertical axis represents Hub gene, the node color represents correlation, and the node size represents significance level.

The CIBERSORT algorithm was further applied to analyse the infiltrated abundance of 22 types of immune cells in patients in the two clusters. The landscape of immune cell infiltration showed that monocytes and neutrophils were predominant among the 22 immune cell types in those with CHD (Figure 9A). Compared with the patients in Cluster 1, the proportion of infiltrated M0 macrophages and plasma cells were lower in Cluster 2 (Figure 9B). The correlation results showed that the hub genes were positively correlated with macrophages (Figure 9C) in patients in Cluster 1, and negatively correlated with follicular helper T cells and Tregs (Figure 9D) in patients in Cluster 2. These findings demonstrated a strong link between m6A RNA modification and immune cell infiltration in patients with CHD, thus indicating that this type of modification further functions in the development and progression of CHD.

### The related hub genes were differentially expressed in CHD

**Figure 10:**
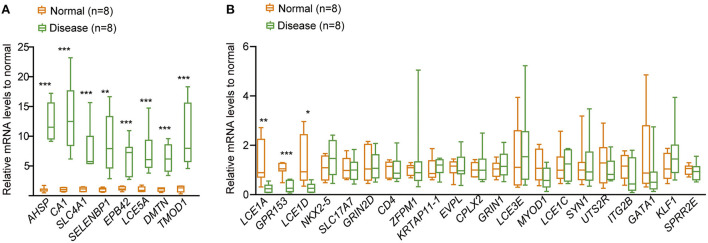
qRT-PCR to detect the relative expression of the 30 hub genes in CHD. **(A)** The relative expressional levels of the eight up-regulated hub genes in CHD. **(B)** The relative expressional levels of the three down-regulated hub genes and genes that were not changed in CHD compared to the normal people. The statistical significance was calculated *via* one-way *ANOVA*, ***P < 0.001, **P < 0.01, *P < 0.05.

To verify the potential role of m6A RNA modification in the development of CHD, the whole blood cells isolated from patients (*n* = 8) with CHD piror to undergoing cardiac catheterization and normal people (*n* = 8) were collected, and the mRNA expression levels of the related hub genes in the patients with CHD were detected by qRT-PCR. The result showed that eight hub genes were upregulated in patients with CHD compared to the normal people (Figure 10A), including alpha hemoglobin stabilizing protein (*AHSP*), carbonic anhydrase 1 (*CA1*), solute carrier family 4 member 1 (*SLC4A1*), selenium binding protein 1 (*SELENBP1*), erythrocyte membrane protein band 4.2 (*EPB42*), tropomodulin 1 (*TMOD1*), late cornified envelope 5A (*LCE5A*), dematin actin binding protein (*DMTN*). In addition, three hub genes were downregulated in CHD patients, and they were late cornified envelope 1A (*LCE1A*), G protein-coupled receptor 153 (*GPR153*), late cornified envelope 1D (*LCE1D*) (Figure 10B). This indicated that these genes may have critical roles in the development of CHD, thus further confirming the potential regulatory role of m6A RNA modification in CHD progression.

## Discussion

m6A RNA methylation, in which a methyl group is added to the nitrogenous base at the sixth position of the adenine residue in RNA, has emerged as one of the most common internal modifications of RNAs (63) and is linked to multiple human diseases, including CVD (3, 20, 21, 64, 65), chronic obstructive pulmonary disease (66), neurodegenerative disease (67), periodontitis (18), cancers (68), and metabolic syndromes (69). The so-called “writer,” “eraser,” and “reader” proteins can respectively add, remove, or recognize m6A-modified sites and impact important biological processes (70, 71). The discovery of these regulators increased our perception of the function of the m6A RNA modification.

According to LASSO regression, the current study first identified four m6A regulators that were significant in the development and progression of CHD, including three m6A “readers” (*HNRNPC, YTHDC2*, and *YTHDF3*) and one “writer” (*ZC3H13*) among the 30 differentially expressed m6A regulators in CHD samples. Compared with previous models (62), LASSO regression is characterized by its use of multidimensional continuous dependent variables to build linear models. LASSO regression requires minimal data formatting and is widely used. Additionally, LASSO regression can also screen variables to reduce the model complexity and selectively include variables in the model to obtain better performance parameters, and the complexity of the model can be controlled by a series of parameters to avoid overfitting. Based on the expression of the four genes, CHD patients had significantly higher risk scores than healthy people. Meanwhile, the expression levels of the four m6A regulators, especially that of *YTHDC2*, were predictive of high CHD risk. The results indicate that these genes might be related to the prognoses of CHD patients and could play prodevelopmental roles in CHD. As an m6A “reader,” *HNRNPC* belongs to the subfamily of ubiquitously expressed heterogeneous nuclear ribonucleoproteins (hnRNPs) and is implicated in oncogenic functions in various tumor (72). CHD is associated with a higher incidence of brain pathologies. For example, depression and CHD are highly comorbid conditions. A new study showed that brain-derived neurotrophic factor (BDNF) plays an important role in cardiovascular processes (73). HNRNPC was recently shown to have enriched binding sites in the brain (74). This strengthens our finding that *HNRNPC* is a potential biomarker and prognostic signature. YTHDC2 belongs to the YT521-B homology superfamily, contains the YTH domain, which is typical of eukaryotes. The protein has a conserved m6A-binding domain (75) and preferentially binds to m6A-modified RNA, modulates the stability of m6A-containing mRNAs and facilitates their efficient translation (75). Recent studies have shown that YTHDC2 can improve the translation efficiency of hypoxia-inducible factor-1alpha (HIF-1a) mRNA (76). HIF-1a has been shown to have the potential function of promoting coronary collateral formation, and might be helpful in predicting the prognoses of patients with CHD (77). Moreover, the important role of hypoxia in CAD development has been reported in previous papers (78). Similar to YTHDC2, YTHDF3 is also a member of the YTH superfamily, and it works closely with other readers to influence the metabolism of m6A-modified mRNA (79, 80). ZC3H13 is an m6A “writer” that facilitates nuclear localization of the writer complex (81). In addition, we found that *YTHDF3* and *ZC3H13* were chromosomally located close to CHD susceptibility genes, indicating that they had potential regulatory effects on CHD development. Although these genes have been known for many years, this is the first study to demonstrate the importance of these four m6A regulators in the development and progression of CHD.

Consensus cluster analysis using the 30 m6A regulators divided the CHD patient samples into two distinct clusters with different m6A regulator expression levels, indicating that two totally distinct m6A modification patterns occur in patients with CHD. A total of 491 genes were found to be significantly upregulated in the samples in Cluster 1 compared with those in Cluster 2, suggesting that these 491 genes might be the target genes for regulation by m6A RNA modifications. Subsequent GO analysis showed that these genes were enriched in the following biological processes: epidermal cell differentiation, keratinocyte differentiation, keratinization, and skin development signaling pathways. Among the 491 m6A RNA modification-related DEGs, 30 were identified as hub genes by PPI network analysis. These hub genes were enriched in the following biological processes: keratinization, chromatin, and sequence-specific double-stranded DNA binding. The GO results suggested that keratinization, epidermal cell differentiation, and skin development signaling pathways may be the common bioprocesses that influence m6A RNA modification in CHD. Skin lesions are frequent manifestations of underlying systemic conditions, including systemic autoimmune vasculitis (82). For example, patients with psoriasis are up to 50% more likely to develop CVD, and this CVD risk increases with skin symptom severity (83). Our results showed that the dysregulation of keratinization and skin development may be a result of m6A RNA modification disorder. This may be a pathway by which m6A RNA modification impacts CHD development. Moreover, LCE1A protein was ranked as the top protein playing a key role in CHD, which was found to be downregulated in CHD by qRT-PCR. LCE1A belongs to the LCE gene cluster within the EDC on chromosome 1. The LCE cluster contains multiple conserved genes that encode stratum corneum proteins, and these genes are expressed relatively late during fetal assembly of the skin cornified envelope (84). These results all indicate that the dysregulation of m6A RNA modification impacts biological processes in patients with CHD that ultimately influence the development of this disease.

Notably, we found that many miRNAs were closely related to the hub genes that were affected by m6A regulators. For example, hsa-mir-146a-5p was the most common miRNA and was linked to eight target hub genes. Sang J's team found that hsa-mir-146a-5p was regulated by the m6A “writer” *METTL14* to function in cancer invasion and migration (85). In addition, hsa-miR-146a-5p was reported to be involved in the innate immune response by regulating IFN-β signaling (86). The miRNA–mRNA network constructed in this study indicates that hsa-miR-146a-5p might be an m6A regulator target that regulates CHD development, although this hypothesis requires further verification. Several miRNA polymorphisms have been associated with susceptibility to specific health disorders, including CHD (87). Several papers have established that miRNAs regulate various cardiovascular development processes and functions, and a deregulated cardiac-enriched miRNA profile plays a critical role in the pathogenesis of CHD and biological aging (88). By constructing the competing endogenous RNA (ceRNA) regulation network, 308 miRNAs and 883 lncRNAs were found to be associated with the hub genes. Among these ncRNAs, hsa-miR-6884-5p, hsa-miR-485-5p, hsa-miR-16-5p, hsa-miR-497-5p, and hsa-miR-665 regulate multiple miRNAs and are regulated by various lncRNAs. For example, we found that 107 miRNAs were regulated by lnc-NEAT1. A recent study showed that NEAT1/miR-140-3p/MAPK1 mediates the viability and survival of coronary endothelial cells and affects coronary atherosclerotic heart disease (89). miR-140-3p was identified as a valuable biomarker for risk estimation in CHD to predict mortality in secondary prevention settings (90). miR-140-3p was also found to be the target miRNA of lnc-SNHG1 in this study, with the latter regulated by METTL3 (91). Additionally, we found that miR-140-3p was correlated with six hub genes (*CD4, GRIN1, ZEPM1, SLC17A7, EPB42*, and *SLC4A1*). These findings may explain the potential regulatory effects of m6A RNA modifications on the development of CHD. Based on the mRNAs-TFs network, *GATA1* was found to be prominent and interact with nine TFs and three hub genes. *GATA1* is one of the most meaningful TFs for biologists because it interacts with a large number of other TFs and regulates many haematopoietic genes (92). The common role of *GATA1* in human disease is its procancer function (93). The potential role of this gene in CHD was first revealed in our study and should be further investigated. These findings provide a prospective theoretical basis for further research on CHD.

Emerging evidence has indicated that inflammation and immune activation appear to be important in the pathogenesis of CHD (94). Various types of immune cells play specific roles in the human immune response and are involved in the strong crosstalk network of the immune system (70, 95). Recently, m6A RNA modification has attracted increasing attention in the regulation of the human immune system (96) based on its important role in the pathogenesis and development of human diseases (97). In the present study, we found that the proportion of infiltrated immune cells differed between the two patient clusters constructed based on the m6A regulators. The proportions of 28 types of immune cells were calculated by ssGSEA, and the results showed that patients in Cluster 2 had an activated immune level with higher infiltrating proportions of activated CD4 T cells, activated CD8 T cells, effector memory CD8 T cells, central memory CD4 T cells, regulatory T cells, gamma delta T cells, immature dendritic cells, memory B cells, monocytes, natural killer cells, and type 2 T helper cells, while patients in Cluster 1 had higher infiltrating proportions of natural killer T cells, CD56dim natural killer cells, type 1 T helper cells and type 17 T helper cells. Previous studies showed that the infiltration of CD8+ T cells could enhance the accumulation of macrophages and inflammation in a human disease model (98, 99). CD4+ T cells are subdivided into two main subsets according to their functions: effector cells and regulatory T (Treg) cells (100). The main function of Treg cells is to avoid autoimmune reactions and to stop the effector response against exogenous antigens, when the response itself becomes dangerous to the host (100). Type 1 T helper (Th1) and type 17 T helper (Th17) cells are involved in chronic inflammatory disorders and the pathogeneses of autoimmune diseases, whereas type 2 T helper (Th2) cells play a critical role in allergies (100). Additionally, the CIBERSORT algorithm was applied to analyse the 22 types of infiltrating immune cells. The results showed that the infiltrated proportions of M0 macrophages and plasma cells were lower in the immune microenvironment of CHD patients in Cluster 2 than those of patients in Cluster 1. Macrophages are the primary contributors to potentially pathological inflammatory processes, which produce large numbers of inflammatory cytokines in response to danger signals (101). These cytokines can initiate a cascade of inflammatory mediator release to cause wholesale tissue destruction (101). Both algorithms used in this study showed significant correlations between m6A RNA modification and these proinflammatory lymphocytes, providing evidence that the infiltrating proportions of these lymphocytes were higher in CHD patients in Cluster 2, and these patients also had higher levels of m6A regulators. The enhanced expression of m6A regulators leads to a high level of m6A RNA methylation, which further changes the expression of target genes and ultimately influences the immune microenvironment.

A previous study identified differentially methylated m6A sites within both mRNAs and lncRNAs between CAD and control groups by methylated RNA immunoprecipitation sequencing (MeRIP-seq) (25). This finding indicates that m6A RNA modification plays an important role in the CHD process. Our results are consistent with it. However, our study aimed to analyse distinct clusters based on m6A RNA modifications, including examinations of DEGs and the immune microenvironment. We systematically revealed the heterogeneity of m6A RNA modifications in CHD and indicated the corresponding biological processes influenced in CHD by analyzing DEGs between the distinct clusters. This study provides a novel theoretical basis for revealing the pathogenesis of CHD regulated by m6A regulators. It also provides a prospective theoretical basis for further studies on the regulatory pathway of m6A RNA modification. Moreover, the influence of CHD-related m6A RNA modification on immune system disorders was clarified for the first time, thus providing a potentially significant research foundation for elucidating the pathogenesis of CHD. Finally, many target miRNAs and TFs regulated by m6A RNA modifications were identified in this study. These results will provide multiple potential approaches for further studies on the mechanisms of m6A modification.

However, this study had certain limitations. First, due to the lack of disease-related prognostic indices in the GEO datasets, the clinical indicators of CHD were not analyzed. Therefore, a large amount of clinical information from CHD patients should be collected to construct a validation set, and additional clinical information should be collected through follow-ups to further analyse the relationships between m6A modification and clinical parameters in CHD patients. The effect of m6A RNA modification on the prognosis of CHD needs to be further explained. Second, *in vivo* and *in vitro* experiments were performed to further explain the specific roles of the four significant m6A regulators in the development of CHD. Additionally, methylated RNA immunoprecipitation sequencing (MeRIP-seq) may be conducted to identify the m6A RNA modification patterns in CHD and to identify the significant genes involved in m6A modification. Finally, we found 308 miRNAs that were closely related to the m6A-related DEGs. These miRNAs might be linked to CHD in future analyses.

In conclusion, the current study suggests that changes in m6A RNA modification patterns mediated by regulators are involved in the immune microenvironment of patients with CHD and play significant roles in the progression of the disease. These findings provide potential novel biomarkers for the diagnosis of CHD.

## Data availability statement

The datasets presented in this study can be found in online repositories. The names of the repository/repositories and accession number(s) can be found in the article/Supplementary materials.

## Ethics statement

The studies involving human participants were reviewed and approved by Ethics Committee of Qingdao Hiser Hospital Affiliated to Qingdao University. The patients/participants provided their written informed consent to participate in this study.

## Author contributions

ZL, TJ, and YC designed experiments and interpreted data. ZL, YS, MW, RS, KQ, YZ, and TJ conducted bioinformatic and statistical analyses. ZL wrote the paper. All authors have read and approved the manuscript for publication.

## Funding

This work was supported by grants from the Natural Science Foundation of Shandong Province [ZR2021MH253 to YC].

## Conflict of interest

The authors declare that the research was conducted in the absence of any commercial or financial relationships that could be construed as a potential conflict of interest.

## Publisher's note

All claims expressed in this article are solely those of the authors and do not necessarily represent those of their affiliated organizations, or those of the publisher, the editors and the reviewers. Any product that may be evaluated in this article, or claim that may be made by its manufacturer, is not guaranteed or endorsed by the publisher.
