# Supplementary material for: m6A regulator-mediated RNA methylation modification patterns are involved in immune microenvironment regulation of coronary heart disease
Source: Front Cardiovasc Med. 2022 Aug 25;9:905737. doi: 10.3389/fcvm.2022.905737 (PMC9453453; doi:10.3389/fcvm.2022.905737)
Supplement: Supplementary file 1 [file Image_1.pdf]

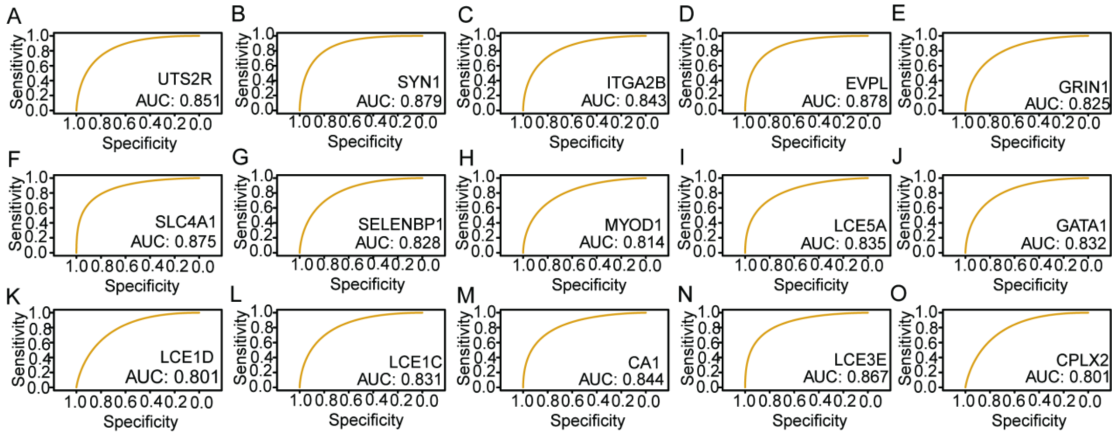

Supplementary Figure 1 ROC analysis of the hub-genes in CHD.

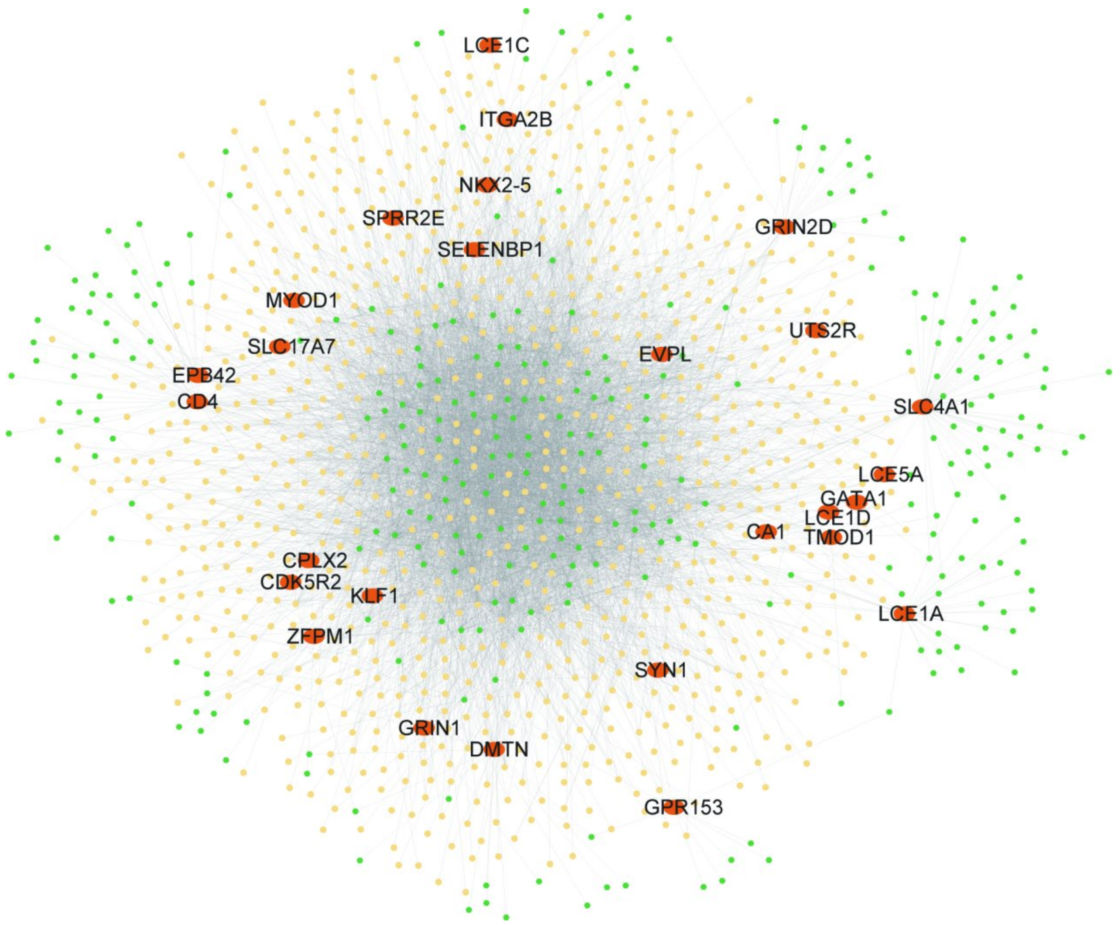

Supplementary Figure 2 The mRNA-lncRNA-miRNA interaction network. The orange label represents the mRNAs we selected, the green label represents miRNAs, and the yellow label represents lncRNAs.
