# Supplementary material for: m6A regulator-mediated RNA methylation modification patterns are involved in immune microenvironment regulation of coronary heart disease
Source: Front Cardiovasc Med. 2022 Aug 25;9:905737. doi: 10.3389/fcvm.2022.905737 (PMC9453453; doi:10.3389/fcvm.2022.905737)
Supplement: Supplementary file 15 [file Data_Sheet_14.docx]

#Figure 1

rm(list=ls())

setwd("D:\\job\\SNOW2021\\3-cad-2.7\\2-geo")

data1<-read.table("D:\\job\\SNOW2021\\3-cad-2.7\\data\\GSE20680_series_matrix.txt",header = T,sep = "\t")

data2<-read.table("D:\\job\\SNOW2021\\3-cad-2.7\\data\\GSE20681_series_matrix.txt",header = T,sep = "\t")

library(GEOquery)

library(dplyr)

GPL4133 <- getGEO("GPL4133")

anno= GPL4133@dataTable@table

anno<-anno[which(anno$ID %in% data1$ID_REF),]

data1$gene=anno$GENE_SYMBOL

data1=aggregate(x=data1,by = data1$gene %>% list(),FUN = mean)#澶氫釜鎺㈤拡瀵瑰簲涓€涓熀鍥犲彇骞冲潎

data2$gene=anno$GENE_SYMBOL

data2=aggregate(x=data2,by = data2$gene %>% list(),FUN = mean)#澶氫釜鎺㈤拡瀵瑰簲涓€涓熀鍥犲彇骞冲潎

rownames(data1)<-data1$Group.1

rownames(data2)<-data2$Group.1

data1<-data1[-1,-c(1,2,198)]

data2<-data2[-1,-c(1,2,201)]

gsms <- paste0("00000000000000000000000000000000000000000000000000",

"00111111111111111111111111111111111111111111111111",

"11111111111111111111111111111111111111111111111111",

"111111111111111111111111111111111111111111111")

sml <- strsplit(gsms, split="")[[1]]

sm <- which(sml != "X")

sml <- sml[sm]

length(which(sml %in% "1")) #143

length(which(sml %in% "0")) #52

data1<-data1[,c(which(sml %in% "1"),which(sml %in% "0"))]

gsms <- paste0("10101010101010101010101010101010101010101010101010",

"10101010101010101010101010101010101010101010101010",

"10101010101010101010101010101010101010101010101010",

"101010101010101010101010101010101010101010101010")

sml <- strsplit(gsms, split="")[[1]]

sm <- which(sml != "X")

sml <- sml[sm]

length(which(sml %in% "1")) #99

length(which(sml %in% "0")) #99

data2<-data2[,c(which(sml %in% "1"),which(sml %in% "0"))]

data3<-read.table("D:\\job\\SNOW2021\\3-cad-2.7\\data\\GSE12288_series_matrix.txt",header = T,sep = "\t")

#绗竴绉嶆柟娉?

GPL96 <- getGEO("GPL96")

anno1=GPL96@dataTable@table

gene=anno1$`Gene Symbol` %>% strsplit(" ///")

gene_symbol=NULL

for(i in 1:length(gene)){

gene_symbol=c(gene_symbol,gene[[i]][1])

}

data3$gene<-gene_symbol

data3=aggregate(x=data3,by = data3$gene %>% list(),FUN = mean)#澶氫釜鎺㈤拡瀵瑰簲涓€涓熀鍥犲彇骞冲潎

rownames(data3)<-data3$Group.1

data3<-data3[,3:224]

gsms <- paste0("11111111111111111111111111111111110010000000001000",

"00010100100010000101101000010000110100101000111101",

"00000000000100101110010000000000100110001001001110",

"10001110001011111101001110001010111101000110111111",

"1111111010010100000111")

sml <- strsplit(gsms, split="")[[1]]

sm <- which(sml != "X")

sml <- sml[sm]

length(which(sml %in% "1")) #112

length(which(sml %in% "0")) #110

data3<-data3[,c(which(sml %in% "0"),which(sml %in% "1"))]

write.table(data3,"test_GSE12288.txt",quote = F,sep = "\t")

aa<-intersect(rownames(data1),rownames(data2))

m6a<-read.csv("D:\\job\\SNOW2021\\3-cad-2.7\\data\\m6a_gene_input.CSV")

bb<-intersect(aa,m6a$m6a.gene) #30

cc<-intersect(rownames(data3),m6a$m6a.gene) #30

data1<-data1[aa,]

data2<-data2[aa,]

count_matrix<-cbind(data1,data2)

count_matrix<-na.omit(count_matrix)

gs <- factor( c(rep("GSE20680",195),rep("GSE20681", 198)))

groups <- make.names(c("GSE20680","GSE20681"))

levels(gs) <- groups

ord <- order(gs)

palette(c( "#E6AB02", "#66A61E", "#A6761D", "#B32424", "#B324B3", "#666666"))

pdf("box1.pdf",width = 9,height =6.6)

par(mar=c(7,4,2,1))

boxplot(count_matrix, boxwex=0.6, notch=T, outline=FALSE, las=2, col=gs[ord],border = gs[ord])

legend("topleft", groups, fill=palette(), bty="n")

dev.off()

library(sva)

batch <- c(rep("1",195),rep("2",198))

adjusted_counts <- ComBat(count_matrix, batch=batch)

pdf("box2.pdf",width =9,height = 6.6)

par(mar=c(7,4,2,1))

boxplot(adjusted_counts,boxwex=0.6,notch=T,outline=FALSE, las=2, col=gs[ord],border = gs[ord])

legend("topleft", groups, fill=palette(), bty="n")

dev.off()

cla<-c(rep("case",143),rep("control",52),rep("case",99),rep("control",99))# 242 151

ord <- order(cla)

adjusted_counts1<-adjusted_counts[,ord]

write.csv(adjusted_counts1,"adjusted_geo.csv")

library(FactoMineR)

library(factoextra)

count_matrix<-as.matrix(t(count_matrix))

cla <- c(rep("GSE20680",195),rep("GSE20681", 198))

iris.pca <- PCA(count_matrix,graph = F)

pdf("pca1.pdf",width = 6,height = 4.5)

ind.p<-fviz_pca_ind(iris.pca,geom.ind = "point", col.ind = cla,

palette = c("#E6AB02", "#66A61E"),

addEllipses = TRUE,

legend.title = "Groups" )

ggpubr::ggpar(ind.p,title = "")

dev.off()

count_matrix1<-as.matrix(t(adjusted_counts))

iris.pca <- PCA(count_matrix1,graph =F)

pdf("pca2.pdf",width = 6,height = 4.5)

ind.p<-fviz_pca_ind(iris.pca,geom.ind = "point", col.ind = cla,

palette = c("#E6AB02", "#66A61E"),

addEllipses = TRUE,

legend.title = "Groups" )

ggpubr::ggpar(ind.p,title = "")

dev.off()

#Figure2

rm(list=ls())

setwd("D:\\job\\SNOW2021\\3-cad-2.7\\3-m6a")

data<-read.csv("D:\\job\\SNOW2021\\3-cad-2.7\\2-geo\\adjusted_geo.csv")

rownames(data)<-data$X

data<-data[,-1]

data<-log2(data)

m6a<-read.csv("D:\\job\\SNOW2021\\3-cad-2.7\\data\\m6a_gene_input.CSV")

bb<-intersect(rownames(data),m6a$m6a.gene)

library(RCircos)

library(dplyr)

chr <- read.csv("chromosome_locate_input.csv")

wri<-c("METTL3","METTL14","WTAP","VIRMA","ZC3H13","CBLL1","RBM15","RBM15B","METTL16","ZCCHC4","PCIF1")

era<-c("FTO","ALKBH3","ALKBH5")

rea<-setdiff(bb,union(wri,era))

chr_wri<-chr[which(chr$Gene %in% wri),]

pdf(width =6,height =6,file = "chromosome_wri.pdf")

data(UCSC.HG38.Human.CytoBandIdeogram)

cyto.info <- UCSC.HG38.Human.CytoBandIdeogram

RCircos.Set.Core.Components(cyto.info)

RCircos.Set.Plot.Area()

RCircos.Chromosome.Ideogram.Plot()

RCircos.Gene.Connector.Plot(chr_wri, track.num = 1, side = "in")

RCircos.Gene.Name.Plot(chr_wri, name.col=4,track.num=2, side="in")

dev.off()

chr_era<-chr[which(chr$Gene %in% era),]

pdf(width =6,height =6,file = "chromosome_era.pdf")

data(UCSC.HG38.Human.CytoBandIdeogram)

cyto.info <- UCSC.HG38.Human.CytoBandIdeogram

RCircos.Set.Core.Components(cyto.info)

RCircos.Set.Plot.Area()

RCircos.Chromosome.Ideogram.Plot()

RCircos.Gene.Connector.Plot(chr_era, track.num = 1, side = "in")

RCircos.Gene.Name.Plot(chr_era, name.col=4,track.num=2, side="in")

dev.off()

chr_rea<-chr[which(chr$Gene %in% rea),]

pdf(width =6,height =6,file = "chromosome_rea.pdf")

data(UCSC.HG38.Human.CytoBandIdeogram)

cyto.info <- UCSC.HG38.Human.CytoBandIdeogram

RCircos.Set.Core.Components(cyto.info)

RCircos.Set.Plot.Area()

RCircos.Chromosome.Ideogram.Plot()

RCircos.Gene.Connector.Plot(chr_rea, track.num = 1, side = "in")

RCircos.Gene.Name.Plot(chr_rea, name.col=4,track.num=2, side="in")

dev.off()

#Figure 3

setwd("D:\\job\\SNOW2021\\3-cad\\4-m6a-deg")

data<-read.csv("D:\\job\\SNOW2021\\3-cad\\2-geo\\adjusted_geo.csv")

rownames(data)<-data$X

data<-data[,-1]

m6a<-read.csv("D:\\job\\SNOW2021\\3-cad\\data\\m6a_gene_input.CSV")

bb<-intersect(rownames(data),m6a$m6a.gene)

library(VennDiagram)

venn.plot<-venn.diagram(list('genes'=rownames(data),"m6A"=m6a$m6a.gene), filename = NULL,

lty=1,lwd=1,col="black",fill=c("#E6AB02", "#66A61E"),alpha=0.60,cat.col="#333333", units = "in", cat.cex=0.8, cat.fontface="bold", margin=0.07, cex=0.8,scale=F)

ggsave(venn.plot, file="m6a-venn.pdf",width = 5,height = 3)

data_1<-data[which(rownames(data) %in% m6a$m6a.gene),]

write.table(data_1,"m6a_exp.txt",row.names = T,quote = F,sep = "\t")

library(ggplot2)

library(ggthemes)

library(ggpubr)

wri<-c("METTL3","METTL14","WTAP","VIRMA","ZC3H13","CBLL1","RBM15","RBM15B","METTL16","ZCCHC4","PCIF1")

era<-c("FTO","ALKBH3","ALKBH5")

data1<-as.data.frame(t(data[which(rownames(data) %in% c(wri)),]))

data2<-as.data.frame(t(data[which(rownames(data) %in% c(era)),]))

data1<-cbind(data1,data2)

library(Hmisc)

library("Rmisc")

library("plyr")

colnames(data1)

a<-c(rep("case",242),rep("control",151))# 242 151

library(Rmisc)

p <- ggplot(data1, aes(CBLL1,ALKBH5)) + geom_point(aes(colour=factor(a))) + #娣诲姞鐐癸紝鎸夌収鎬у埆浣跨敤涓嶅悓鐨勯鑹?

stat_smooth(method=lm)+ theme_bw()+ scale_colour_manual(values=c("#E6AB02", "#66A61E"))+

theme(legend.position="none")+stat_cor(data=data1, method = "pearson") #鍒犻櫎鍥炬敞

p1<-ggExtra::ggMarginal(p, type = "densigram", xparams=list(fill = "#E6AB02"), yparams = list(fill="#66A61E"))

p <- ggplot(data1, aes(ZC3H13,ALKBH5)) + geom_point(aes(colour=factor(a))) + #娣诲姞鐐癸紝鎸夌収鎬у埆浣跨敤涓嶅悓鐨勯鑹?

stat_smooth(method=lm)+ theme_bw()+ scale_colour_manual(values=c("#E6AB02", "#66A61E"))+

theme(legend.position="none")+stat_cor(data=data1, method = "pearson") #鍒犻櫎鍥炬敞

p2<-ggExtra::ggMarginal(p, type = "densigram", xparams=list(fill = "#E6AB02"), yparams = list(fill="#66A61E"))

p <- ggplot(data1, aes(ZCCHC4,ALKBH5)) + geom_point(aes(colour=factor(a))) + #娣诲姞鐐癸紝鎸夌収鎬у埆浣跨敤涓嶅悓鐨勯鑹?

stat_smooth(method=lm)+ theme_bw()+ scale_colour_manual(values=c("#E6AB02", "#66A61E"))+

theme(legend.position="none")+stat_cor(data=data1, method = "pearson") #鍒犻櫎鍥炬敞

p3<-ggExtra::ggMarginal(p, type = "densigram", xparams=list(fill = "#E6AB02"), yparams = list(fill="#66A61E"))

p <- ggplot(data1, aes(WTAP,ALKBH5)) + geom_point(aes(colour=factor(a))) + #娣诲姞鐐癸紝鎸夌収鎬у埆浣跨敤涓嶅悓鐨勯鑹?

stat_smooth(method=lm)+ theme_bw()+ scale_colour_manual(values=c("#E6AB02", "#66A61E"))+

theme(legend.position="none")+stat_cor(data=data1, method = "pearson") #鍒犻櫎鍥炬敞

p4<-ggExtra::ggMarginal(p, type = "densigram", xparams=list(fill = "#E6AB02"), yparams = list(fill="#66A61E"))

p <- ggplot(data1, aes(RBM15B,ALKBH5)) + geom_point(aes(colour=factor(a))) + #娣诲姞鐐癸紝鎸夌収鎬у埆浣跨敤涓嶅悓鐨勯鑹?

stat_smooth(method=lm)+ theme_bw()+ scale_colour_manual(values=c("#E6AB02", "#66A61E"))+

theme(legend.position="none")+stat_cor(data=data1, method = "pearson") #鍒犻櫎鍥炬敞

p5<-ggExtra::ggMarginal(p, type = "densigram", xparams=list(fill = "#E6AB02"), yparams = list(fill="#66A61E"))

p <- ggplot(data1, aes(RBM15,ALKBH5)) + geom_point(aes(colour=factor(a))) + #娣诲姞鐐癸紝鎸夌収鎬у埆浣跨敤涓嶅悓鐨勯鑹?

stat_smooth(method=lm)+ theme_bw()+ scale_colour_manual(values=c("#E6AB02", "#66A61E"))+

theme(legend.position="none")+stat_cor(data=data1, method = "pearson") #鍒犻櫎鍥炬敞

p6<-ggExtra::ggMarginal(p, type = "densigram", xparams=list(fill = "#E6AB02"), yparams = list(fill="#66A61E"))

p <- ggplot(data1, aes(PCIF1,ALKBH5)) + geom_point(aes(colour=factor(a))) + #娣诲姞鐐癸紝鎸夌収鎬у埆浣跨敤涓嶅悓鐨勯鑹?

stat_smooth(method=lm)+ theme_bw()+ scale_colour_manual(values=c("#E6AB02", "#66A61E"))+

theme(legend.position="none")+stat_cor(data=data1, method = "pearson") #鍒犻櫎鍥炬敞

p7<-ggExtra::ggMarginal(p, type = "densigram", xparams=list(fill = "#E6AB02"), yparams = list(fill="#66A61E"))

p <- ggplot(data1, aes(METTL3,ALKBH5)) + geom_point(aes(colour=factor(a))) + #娣诲姞鐐癸紝鎸夌収鎬у埆浣跨敤涓嶅悓鐨勯鑹?

stat_smooth(method=lm)+ theme_bw()+ scale_colour_manual(values=c("#E6AB02", "#66A61E"))+

theme(legend.position="none")+stat_cor(data=data1, method = "pearson") #鍒犻櫎鍥炬敞

p8<-ggExtra::ggMarginal(p, type = "densigram", xparams=list(fill = "#E6AB02"), yparams = list(fill="#66A61E"))

ggsave(p1,file="ALKBH5_1.pdf",width =6,height =4.4)

ggsave(p2,file="ALKBH5_2.pdf",width =6,height =4.4)

ggsave(p3,file="ALKBH5_3.pdf",width =6,height =4.4)

ggsave(p4,file="ALKBH5_4.pdf",width =6,height =4.4)

ggsave(p5,file="ALKBH5_5.pdf",width =6,height =4.4)

ggsave(p6,file="ALKBH5_6.pdf",width =6,height =4.4)

ggsave(p7,file="ALKBH5_7.pdf",width =6,height =4.4)

ggsave(p8,file="ALKBH5_8.pdf",width =6,height =4.4)

rm(list = ls())

setwd("D:\\job\\SNOW2021\\3-cad\\5-forest")

data<-read.csv("D:\\job\\SNOW2021\\3-cad\\2-geo\\adjusted_geo.csv")

rownames(data)<-data$X

data<-data[,-1]

m6a<-read.csv("D:\\job\\SNOW2021\\3-cad\\data\\m6a_gene_input.CSV")

bb<-intersect(rownames(data),m6a$m6a.gene)

library(limma)

library(xlsx)

library(dplyr)

library(DESeq2)

data1<-data[bb,]

data1<-round(2^data1-1)

trait=data.frame(condition=c(rep('c1',242),rep('c2',151)) %>% as.factor())

rownames(trait) <- colnames(data1)

DE<- DESeqDataSetFromMatrix(countData=data1,colData=trait,design = ~condition)

DE <- DESeq(DE)

result <- results(DE, contrast=c("condition","c1","c2"))

head(result)

#鎻愬彇宸紓鍒嗘瀽缁撴灉

result <- result[order(result$pvalue),] # 鎸夌収padj鐨勫ぇ灏忓皢result閲嶆柊鎺掑垪

diff_gene_deseq2 <- subset(result,) %>% as.data.frame()

write.table(diff_gene_deseq2[,1:5],"m6a_deseq2.txt",sep = "\t",quote = F,row.names = T)

group_list<-c(rep('c1',242),rep('c2',151))

group_list = factor(group_list)

design <- model.matrix(~0+group_list)

rownames(design) = colnames(data1)

colnames(design) <- levels(group_list)

contrast.matrix<-makeContrasts(paste0(unique(group_list),collapse = "-"),levels = design)

fit <- lmFit(data1,design)

fit2 <- contrasts.fit(fit, contrast.matrix) ##

fit2 <- eBayes(fit2) ## default no trend !!!

tempOutput = topTable(fit2, coef=1, n=Inf)

tT3 = na.omit(tempOutput)

tT3$id<-rownames(tT3)

write.table(tT3,"cluster-limma.txt" ,col.names = T,row.names = T,sep = "\t",quote = F)

data1<-data[bb,]

cla<-c(rep('c1',242),rep('c2',151))

library(glmnet)

library(dplyr)

fit=glmnet(data1 %>% as.matrix() %>% t(),cla,family = "binomial",alpha = 1)

pdf(file = "1-lasso.pdf",width = 9,height =6.6)

plot(fit, xvar="lambda", label=TRUE,lwd=4)

dev.off()

cvfit=cv.glmnet(data1 %>% as.matrix() %>% t(),cla,family = "binomial")

pdf(file = "2-lambda.pdf",width = 9,height =6.6)

plot(cvfit)

dev.off()

cvfit$lambda.min

coef.min=coef(cvfit,s="lambda.min")

coef.min

coef.min=as.matrix(coef.min)

coef.min.re=coef.min[coef.min!=0,]

coef.min.re <- round(coef.min.re[-1],digits = 3) %>% as.data.frame()

colnames(coef.min.re) <- "Coef"

library(forestplot)

coef.min.re$Gene <- rownames(coef.min.re)

coef.min.re$Sample <- c("n=242 (case=151)")

coef.min.re$'Mean expression' <- data1[rownames(coef.min.re),] %>% apply(1,mean) %>% round(digits = 3)

coef.min.re$mean <- data1[rownames(coef.min.re),] %>% apply(1,mean) %>% round(digits = 3)

coef.min.re$lower <-data1[rownames(coef.min.re),] %>% apply(1,min) %>% round(digits = 3)

coef.min.re$upper <- data1[rownames(coef.min.re),] %>% apply(1,max) %>% round(digits = 3)

coef.min.re2 <- rbind(colnames(coef.min.re),coef.min.re)

coef.min.re2<-coef.min.re2[,c(2,3,1,4,5,6,7)]

pdf(file = "3-forest.pdf",width = 12,height = 9)

forestplot(labeltext = coef.min.re2[,1:4] %>% as.matrix(),

mean = c(NA,coef.min.re$mean), #璁剧疆鍧囧€?

lower = c(NA,coef.min.re$lower), #璁剧疆鍧囧€肩殑low limits

upper = c(NA,coef.min.re$upper), #璁剧疆鍧囧€肩殑up limits

is.summary=c(T,F,F,F,F,F,F,F,F,F,F,F,F,F,F,F,F,F,F,F,F,F,F,F,F,F,F,F,F,F,F,F,F,F,F,F,F,F,F),

zero = 0,

hrzl_lines =list("2"=gpar(lty=2)),

new_page=F,

boxsize = 0.35,

lwd.xaxis=3,

align=c("c"),

col=fpColors(box='#E6AB02',lines = 'black'),

graph.pos = 5)

dev.off()

library(pROC)

gene<-coef.min.re2$Gene[2:5]

coef<-as.numeric(coef.min.re2$Coef[2:5])

data2 <-as.data.frame( t(data1[gene,]))

for (i in 1:nrow(data2)) {

s<-0

for (j in 1:4) {

s<-s+data2[i,j]*coef[j]

}

data2[i,5]<-s

}

data2$cla<-c(rep('c1',242),rep('c2',151))

colnames(data2)[5] <- "score"

roc <- roc(data2$cla,data2$score %>% as.numeric(),ci=T,smooth = F)

pdf(file = "4-ROC.pdf",width = 6,height =4)

plot(roc)

plot(roc, col="#E6AB02", add=T,grid=0.15,main="ROC Curve",cex.main=1.5)

legend(x=0.4,y=0.3,"AUC:0.60",bty = "n",text.col = "black",cex = 1.5)

legend(x=0.4,y=0.2,"95% CI: 0.55-0.66",bty = "n",text.col = "black",cex = 1.5)

dev.off()

data2 <-as.data.frame( t(data[gene,]))

data2$cla<-c(rep('c1',242),rep('c2',151))

library(pROC)

for (i in 1:length(gene)) {

roc1<- roc(data2$cla,data2[,i],smooth =T,ci=F)

aa<-paste0(gene[i],".pdf")

pdf(aa,width = 6,height = 5)

plot(roc1,main=gene[i])

plot(roc1, col="#E6AB02",print.auc=TRUE, add=TRUE, grid=c(0.1, 0.2), print.auc.x=0.45,print.auc.y=0.1)

dev.off()

}

data2 <-as.data.frame( t(data[gene,]))

for (i in 1:nrow(data2)) {

s<-0

for (j in 1:4) {

s<-s+data2[i,j]*coef[j]

}

data2[i,5]<-s

}

data2$cla<-c(rep('c1',242),rep('c2',151))

colnames(data2)[5] <- "score"

data2$score<--data2$score

write.table(data2,"data-score.txt",col.names = T,row.names = T,sep = "\t",quote = F)

test<-read.table("D:\\job\\SNOW2021\\3-cad\\data\\GSE12288_series_matrix.txt",header = T)

rownames(test)<-test$ID_REF

test<-test[,-1]

library(GEOquery)

GPL96<-getGEO("GPL96")

anno= GPL96@dataTable@table

library(dplyr)

gene=anno$`Gene Symbol` %>% strsplit(" /// ")

gene_symbol=NULL

for(i in 1:length(gene)){

gene_symbol=c(gene_symbol,gene[[i]][1])

}

test=test[anno$ID,]

test=aggregate(x=test,by = gene_symbol %>% list(),FUN = mean)#澶氫釜鎺㈤拡瀵瑰簲涓€涓熀鍥犲彇骞冲潎

rownames(test)<-test$Group.1

test<-test[,-1]

gsms <- paste0("00000000000000000000000000000000001101111111110111",

"11101011011101111010010111101111001011010111000010",

"11111111111011010001101111111111011001110110110001",

"01110001110100000010110001110101000010111001000000",

"0000000101101011111000")

sml <- strsplit(gsms, split="")[[1]]

test1<-cbind(test[,which(sml %in% "1")],test[,which(sml %in% "0")])

test1<-test[which(rownames(test) %in% coef.min.re$Gene),]

coef.min.re<-coef.min.re[which(coef.min.re$Gene %in% rownames(test1)),]

coef<-as.numeric(coef.min.re$Coef)

data2 <-as.data.frame( t(test1))

data2<-log2(data2+1)

for (i in 1:nrow(data2)) {

s<-0

for (j in 1:4) {

s<-s+data2[i,j]*coef[j]

}

data2[i,5]<-s

}

data2$cla<-c(rep('c1',length(which(sml %in% "1"))),rep('c2',length(which(sml %in% "0"))))

colnames(data2)[5] <- "score"

data2$score<--data2$score

write.table(data2[,1:5],"data-score-verify.txt",col.names = T,row.names = T,sep = "\t",quote = F)

roc <- roc(data2$cla,data2$score %>% as.numeric(),ci=T,smooth = F)

pdf(file = "D:\\job\\SNOW2021\\3-cad\\5-forest\\5-ROC.pdf",width = 6,height =4)

plot(roc)

plot(roc, col="#E6AB02", add=T,grid=0.15,main="ROC Curve",cex.main=1.5)

legend(x=0.4,y=0.3,"AUC:57%",bty = "n",text.col = "black",cex = 1.5)

dev.off()

rm(list = ls())

setwd("D:\\job\\SNOW2021\\3-cad-2.7\\6-nomo")

data<-read.csv("D:\\job\\SNOW2021\\3-cad-2.7\\2-geo\\adjusted_geo.csv")

rownames(data)<-data$X

data<-data[,-1]

m6a<-read.csv("D:\\job\\SNOW2021\\3-cad-2.7\\data\\m6a_gene_input.CSV")

bb<-intersect(rownames(data),m6a$m6a.gene)

library(ggplot2)

library(ggthemes)

library(ggpubr)

data<-as.data.frame(t(data[,]))

data$cla<-c(rep('c1',242),rep('c2',151))

colnames(data)

phon<-read.table("D:\\job\\SNOW2021\\3-cad-2.7\\5-forest\\data-score.txt",header = T)

phon$cla<-c(rep('c1',242),rep('c2',151))

library(rms)

fit <- lrm(cla~score, data=phon, x=T, y=T)

dc<-datadist(phon)

options(datadist="dc")

nom <- nomogram(fit,fun=plogis,fun.at =c(0.5,1) ,funlabel=c("Risk of CAD"))

pdf(width = 12,height = 8,file = "nomogram-1.pdf",onefile = F)

plot(nom,cex.axis=1.5,cex.var=1.5)

dev.off()

colnames(phon)

fit <- lrm(cla~HNRNPC+YTHDC2+YTHDF3+ZC3H13, data=phon, x=T, y=T)

dc<-datadist(phon)

options(datadist="dc")

nom <- nomogram(fit,fun=plogis,fun.at =c(0.5,1) ,funlabel=c("Risk of CAD"))

pdf(width = 12,height = 8,file = "nomogram-2.pdf",onefile = F)

plot(nom,cex.axis=1.5,cex.var=1.5)

dev.off()

colnames(phon)

phon$cla<-c(rep('0',242),rep('1',151))

phon$cla <- phon$cla%>% as.numeric()

glm <- glm(cla~HNRNPC+YTHDC2+YTHDF3+ZC3H13,data=phon,family = binomial(link = "logit"))

P1 <- predict(glm,type = 'response')

pdf(file = "nomogram_val.pdf",width = 12,height = 8)

val.prob(P1,phon$cla,cex = 1.5)

dev.off()

library(ggDCA)

library(DecisionCurve)

simple<- decision_curve(cla~ score,data = phon, family = binomial(link ='logit'),

thresholds= seq(0,1, by = 0.01),

confidence.intervals =0.95,study.design = 'case-control',

population.prevalence = 0.3)

complex<-decision_curve(cla~HNRNPC+YTHDC2+YTHDF3+ZC3H13,data = phon,

family = binomial(link ='logit'), thresholds = seq(0,1, by = 0.01),

confidence.intervals= 0.95,study.design = 'case-control',

population.prevalence= 0.3)

List<- list(simple,complex)

pdf(width = 12,height = 8,file = "DAC.pdf")

plot_decision_curve(List,curve.names= c('simple','complex'),

cost.benefit.axis =FALSE,col = c("#E6AB02", "#66A61E"),

confidence.intervals =FALSE,standardize = FALSE)

dev.off()

#figure 4

rm(list = ls())

setwd("D:\\job\\SNOW2021\\3-cad\\7-subgroup")

data<-read.csv("D:\\job\\SNOW2021\\3-cad\\2-geo\\adjusted_geo.csv")

rownames(data)<-data$X

data<-data[,-1]

m6a<-read.csv("D:\\job\\SNOW2021\\3-cad\\data\\m6a_gene_input.CSV")

bb<-intersect(rownames(data),m6a$m6a.gene)

library(ggplot2)

library(ggthemes)

library(ggpubr)

data<-as.data.frame(data[bb,1:242])

write.table(data,"m6a_cad_exp.txt",row.names = T,sep = "\t",quote = F)

data1<-data[,]

library(ConsensusClusterPlus)

cluster <- ConsensusClusterPlus(d=data1 %>% as.matrix(), maxK = 10, reps=100, pItem=1, pFeature=1, clusterAlg='pam',title='consensus_cluster',innerLinkage='average', finalLinkage='average', distance='euclidean',plot="pdf",writeTable=T,verbose=T,corUse='everything')

label <- cluster[[2]][["consensusClass"]] %>% as.data.frame()

colnames(label) <- "cluster"

label$sample<-rownames(label)

label<-label[order(label$cluster),]

data1 <- data1[,rownames(label)]

#鐑浘

library(pheatmap)

length(which(label$cluster %in% "1")) # 83

length(which(label$cluster %in% "2")) # 159

c1<-label$sample[which(label$cluster %in% "1")]

c2<-label$sample[which(label$cluster %in% "2")]

write.table(c1,"c1.txt",row.names = F,col.names = F,quote = F)

write.table(c2,"c2.txt",row.names = F,col.names = F,quote = F)

annotation_col = data.frame(Type = factor(c(rep('cluster1',83),rep('cluster2',159))))

rownames(annotation_col) = colnames(data1)

ann_colors = list(Type = c(cluster1="#E6AB02",cluster2="#66A61E"))

p1<-pheatmap(data1,cluster_col=F,cluster_rows = F,

show_rownames =T,show_colnames = F,

annotation_col=annotation_col,annotation_colors = ann_colors)

ggsave(p1,file="cluster-pheatmap.pdf",width = 8,height = 6)

cb2<-as.data.frame(t(data[,rownames(label)]))

immu<- rep(colnames(cb2),each=nrow(cb2)) #缁勫埆鍙橀噺

immu <- factor(immu) #缁勫埆鍥犲瓙鍖?

a<-c(rep('cluster1',83),rep('cluster2',159))

group <- rep(a,ncol(cb2)) #姣忎釜缁勫埆鐨勪袱涓睘鎬?

group <- factor(group) #灞炴€у洜瀛愬寲

value <- c() #闅忔満璧嬪€?

for (j in 1:ncol(cb2)) { value<-c(value,cb2[,j])}

value<-as.numeric(value)

Data <- data.frame(immu_cell=immu,group=group,value=value) #鐢熸垚鏁版嵁妗?

p<-ggplot(Data,aes(x=immu_cell,y=value,fill=group))+

geom_boxplot(width=0.7,size=0.3,outlier.color = NA)+

theme_bw()+scale_fill_manual(values = c("#E6AB02", "#66A61E"))+

theme(panel.grid = element_blank())+

theme(axis.text.x = element_text(angle = 60,hjust = 1))+

stat_compare_means(symnum.args = list(cutpoints = c(0,0.001, 0.01, 0.05, 1),

symbols = c("***", "**", "*", "ns")),label = "p.signif")+

theme(legend.position = 'top')+xlab('')+ylab('gene expression')+labs(fill='Group')

ggsave(p,file="ma6_barplot.pdf",width = 9,height = 6)

library(FactoMineR)

library(factoextra)

iris.pca <- PCA(t(data1),graph = F)

cla<-c(rep('cluster1',83),rep('cluster2',159))

pdf("pca.pdf",width =6,height =4)

ind.p<-fviz_pca_ind(iris.pca,geom.ind = "point", col.ind = cla,

palette = c("#E6AB02", "#66A61E" ),

legend.title = "cluster" )

ggpubr::ggpar(ind.p,title = "")

dev.off()

#Figure 5 # Figure 6

rm(list = ls())

setwd("D:\\job\\SNOW2021\\3-cad\\8-enrich")

data<-read.csv("D:\\job\\SNOW2021\\3-cad\\2-geo\\adjusted_geo.csv")

rownames(data)<-data$X

data<-data[,-1]

c1<-read.table("D:\\job\\SNOW2021\\3-cad\\7-subgroup\\c1.txt")

c2<-read.table("D:\\job\\SNOW2021\\3-cad\\7-subgroup\\c2.txt")

data<-data[,c(c1$V1,c2$V1)]

write.table(data,"cluster_exp.txt",row.names = T,sep = "\t",quote = F)

library(limma)

library(xlsx)

library(ggplot2)

library(ggpubr)

library(ggthemes)

library(ggrepel)

library(dplyr)

group_list <- c(rep('cluster1',83),rep('cluster2',159))

group_list = factor(group_list)

design <- model.matrix(~0+group_list)

rownames(design) = colnames(data)

colnames(design) <- levels(group_list)

contrast.matrix<-makeContrasts(paste0(unique(group_list),collapse = "-"),levels = design)

fit <- lmFit(data,design)

fit2 <- contrasts.fit(fit, contrast.matrix) ##

fit2 <- eBayes(fit2) ## default no trend !!!

tempOutput = topTable(fit2, coef=1, n=Inf)

tT3 = na.omit(tempOutput)

tT3$id<-rownames(tT3)

write.table(tT3,"cluster-limma.txt" ,col.names = T,row.names = T,sep = "\t",quote = F)

DEGs_tt1<-tT3

DEGs_tt1<-na.omit(DEGs_tt1)

DEGs_tt1$logP<--log10(DEGs_tt1$adj.P.Val )

DEGs_tt1$group<-"not-DEGs"

length(unique( DEGs_tt1[which(DEGs_tt1$adj.P.Val<0.05 & abs(DEGs_tt1$logFC)>log2(1.5)),7]))#491

DEGs_tt1$group[which((DEGs_tt1$adj.P.Val<0.05)&(DEGs_tt1$logFC>log2(1.5)))]="up-regulated"

length(unique( DEGs_tt1[which((DEGs_tt1$adj.P.Val<0.05)&(DEGs_tt1$logFC>log2(1.5))),7]))#491

DEGs_tt1$group[which((DEGs_tt1$adj.P.Val<0.05)&(DEGs_tt1$logFC<(-log2(1.5))))]="down-regulated"

length(unique( DEGs_tt1[which((DEGs_tt1$adj.P.Val<0.05)&(DEGs_tt1$logFC<(-log2(1.5)))),7]))#0

#volcano plot

p<-ggplot(DEGs_tt1,aes(x=logFC,y=logP,colour=group))+

xlab("log2 Fold Change")+ylab("-log10(Adjust P-value)")+

ggtitle("Volcano plot") +

theme(plot.title = element_text(hjust = 0.5))+

geom_point(size=0.8,alpha=0.6)+

scale_color_manual(values =c("grey","red")) +

geom_hline(yintercept=1.3,linetype="longdash",col="grey")+

geom_vline(xintercept=c(-log2(1.5),log2(1.5)),linetype="longdash",col="grey")+

theme_few()+theme(legend.title = element_blank(),plot.title = element_text(hjust = 0.5)) #

ggsave(p,file="Volcano.pdf",width = 8,height = 6)

library(pheatmap)

p0.05<-DEGs_tt1[which(DEGs_tt1$adj.P.Val<0.05& abs(DEGs_tt1$logFC)>log2(1.5)),7]

deg1_exp<-data[which(rownames(data)%in% p0.05),]

write.table(p0.05,"p0.05.txt",col.names =F,row.names = F,sep = "\t",quote = F)

annotation_col = data.frame(Type = factor(c(rep('c1',83),rep('c2',159))))

rownames(annotation_col) = colnames(deg1_exp)

ann_colors = list(patient_group = c(c1="#3399FF",c2="#99FFCC"))

library(pheatmap)

p1<-pheatmap(deg1_exp,cluster_col=F,cluster_rows = T,

show_rownames = F,show_colnames = F,

main = "Pheatmap for DEGs of TCGA",

annotation_col=annotation_col)

ggsave(p1,file="pheatmap.pdf",width = 8,height = 6)

library(clusterProfiler)

library(org.Hs.eg.db)

library(xlsx)

library(ggplot2)

library(GOplot)

library(stringr)

library(enrichplot)

library(limma)

limma<-tT3

genelist<-EC$genelist

david<-EC$david

limma<-limma[,c(7,1:6)]

colnames(limma)<-colnames(genelist)

DEGs_tt1<-tT3

gene1 = unique( DEGs_tt1[which(DEGs_tt1$P.Value<0.05& abs(DEGs_tt1$logFC)>log2(1.5)),7])#gene symbol ID

eg = mapIds(x = org.Hs.eg.db,keys = gene1,keytype = "SYMBOL",column = "ENTREZID")

geneList1<-limma$logFC

names(geneList1)<-eg

geneList1<-sort( geneList1,decreasing = T)

library(ggnewscale)

ego_bp <- enrichGO(OrgDb="org.Hs.eg.db",gene = eg,keyType = "ENTREZID",pvalueCutoff = 0.05,

ont = "BP",readable=TRUE)

write.table(ego_bp@result[which(ego_bp@result$pvalue<0.05),] ,"deg_bp_output.txt",row.names = F,quote = F,sep = "\t")

write.csv(ego_bp@result[which(ego_bp@result$pvalue<0.05),],"deg_bp_output.csv",row.names=F)

p<-enrichplot::cnetplot(ego_bp,circular=F,colorEdge = TRUE, foldChange=geneList1,node_label="category")

ggsave(p,file="go_bp.pdf",width = 15,height =10)

ego_cc <- enrichGO(OrgDb="org.Hs.eg.db",gene = eg,keyType = "ENTREZID",pvalueCutoff = 1,

ont = "CC",readable=TRUE)

write.table(ego_cc@result[which(ego_cc@result$pvalue<0.05),] ,"deg_cc_output.txt",row.names = F,quote = F,sep = "\t")

write.csv(ego_cc@result[which(ego_cc@result$pvalue<0.05),],"deg_cc_output.csv",row.names=F)

p<-enrichplot::cnetplot(ego_cc,circular=F,colorEdge = TRUE ,foldChange=geneList1,node_label="category")

ggsave(p,file="go_cc.pdf",width = 15,height =10)

ego_mf <- enrichGO(OrgDb="org.Hs.eg.db",gene = eg,keyType = "ENTREZID",pvalueCutoff = 0.05,

ont = "MF",readable=TRUE)

write.table(ego_mf@result[which(ego_mf@result$pvalue<0.05),] ,"deg_mf_output.txt",row.names = F,quote = F,sep = "\t")

write.csv(ego_mf@result[which(ego_mf@result$pvalue<0.05),],"deg_mf_output.csv",row.names=F)

p<-enrichplot::cnetplot(ego_mf,circular=F,colorEdge = TRUE ,foldChange=geneList1,node_label="category")

ggsave(p,file="go_mf.pdf",width =15,height =10)

a<-ego_bp@result[which(ego_bp@result$pvalue<0.05),c(1,2,8,5)]

b<-ego_cc@result[which(ego_cc@result$pvalue<0.05),c(1,2,8,5)]

c<-ego_mf@result[which(ego_mf@result$pvalue<0.05),c(1,2,8,5)]

ego_result_ALL<-rbind(a,b,c)

ego_result_ALL$Category<-c(rep("BP",nrow(a)),rep("CC",nrow(b)),rep("MF",nrow(c)))

ego_result_ALL$geneID<-gsub("/",",",ego_result_ALL$geneID)

ego_result_ALL<-ego_result_ALL[,c(5,1,2,3,4)]

colnames(ego_result_ALL)<-colnames(david)

write.table(ego_result_ALL ,"deg_go_output.txt",row.names = F,quote = F,sep = "\t")

write.csv(ego_result_ALL,"deg_go_output.csv",row.names=F)

circ <- circle_dat(ego_result_ALL,limma)

pdf("deg_GO.pdf",width = 12,height = 9)

GOBubble(circ, title = '', colour = c("#E6AB02", "blue", "#66A61E" ), display = 'multiple', labels =3.4,table.legend = F)

dev.off()

#KEGG enrichment

david_m<-read.table("david.txt",header = T,sep = "\t",fill = T,quote = "")

david_m<-david_m[which(david_m$PValue<0.05),]

ekegg_result_ALL<-david_m[which(david_m$Category %in% 'KEGG_PATHWAY'),]

ekegg_result_ALL$ID<-substr(ekegg_result_ALL$Term,1,8)

ekegg_result_ALL$Term<-substr(ekegg_result_ALL$Term,10,nchar(ekegg_result_ALL$Term))

ekegg_result_ALL<-ekegg_result_ALL[,c(1,14,2,6,5)]

colnames(ekegg_result_ALL)<-colnames(david)

write.table(ekegg_result_ALL,"deg_kegg_output.txt",row.names = F,quote = F,sep = "\t")

write.xlsx2(ekegg_result_ALL,"deg_kegg_output.xls",row.names=F)

circ <- circle_dat(ekegg_result_ALL,limma)

geneList1<-limma$logFC

names(geneList1)<-limma$ID

geneList1<-sort( geneList1,decreasing = T)

library("pathview")

hsa04080<- pathview(gene.data = geneList1, pathway.id = "hsa04080",species = "hsa",

limit = list(gene=max(abs(geneList1)), cpd=1))

#gsea_kegg

gmt<-read.gmt("c2.cp.kegg.v7.4.symbols.gmt") #璇籫mt鏂囦欢

gsea<-GSEA(geneList1,TERM2GENE = gmt,pvalueCutoff = 0.05) #GSEA鍒嗘瀽

result1<-gsea@result

gsea@result$Description<-tolower(gsea@result$Description)

library(enrichplot)

#鐗瑰畾閫氳矾浣滃浘

p<-gseaplot2(gsea,1,color="red",pvalue_table = T) # 鎸夌涓€涓仛浜岀淮鐮佸浘锛屽苟鏄剧ずp鍊?

ggsave(p,file="gsea_kegg1.pdf",width =8,height =6)

p<-gseaplot2(gsea,2,color="red",pvalue_table = T) # 鎸夌涓€涓仛浜岀淮鐮佸浘锛屽苟鏄剧ずp鍊?

ggsave(p,file="gsea_kegg2.pdf",width =8,height =6)

p<-gseaplot2(gsea,3,color="red",pvalue_table = T) # 鎸夌涓€涓仛浜岀淮鐮佸浘锛屽苟鏄剧ずp鍊?

ggsave(p,file="gsea_kegg3.pdf",width =8,height =6)

p<-gseaplot2(gsea,4,color="red",pvalue_table = T) # 鎸夌涓€涓仛浜岀淮鐮佸浘锛屽苟鏄剧ずp鍊?

ggsave(p,file="gsea_kegg4.pdf",width =8,height =6)

p<-gseaplot2(gsea,5,color="red",pvalue_table = T) # 鎸夌涓€涓仛浜岀淮鐮佸浘锛屽苟鏄剧ずp鍊?

ggsave(p,file="gsea_kegg5.pdf",width =8,height =6)

write.table(gsea@result,"m1_kegg.txt",row.names = F,col.names = T,sep = "\t",quote = F)

write.csv(gsea@result,"m1_kegg.csv",row.names = F)

gsea1<-gsea@result[gsea@result$enrichmentScore>0,]

gsea1<-gsea1[order(gsea1$p.adjust),]

gsea2<-gsea@result[gsea@result$enrichmentScor<0,]

gsea2<-gsea2[order(gsea2$p.adjust),]

k1<-ggplot(gsea1[,],aes(x=enrichmentScore,y=Description))+

geom_point(aes(size=setSize,color=p.adjust))+

scale_color_gradient(low="#66A61E",high ="#E6AB02")+

labs(color=expression(p.adjust),size="count",x="enrichmentScore",y="")+

theme_bw()

ggsave(k1,file="gsea_kegg1.pdf",width =6,height =7.5)

k1<-ggplot(gsea2[,],aes(x=enrichmentScore,y=Description))+

geom_point(aes(size=setSize,color=p.adjust))+

scale_color_gradient(low="#66A61E",high ="#E6AB02")+

labs(color=expression(p.adjust),size="count",x="enrichmentScore",y="")+

theme_bw()

ggsave(k1,file="gsea_kegg2.pdf",width =6,height =7.5)

#gsea_go

###################################################################

#c5.go.v7.2.entrez

gmt<-read.gmt("c5.go.v7.2.symbols.gmt") #璇籫mt鏂囦欢

gsea<-GSEA(geneList1,TERM2GENE = gmt,pvalueCutoff = 0.05) #GSEA鍒嗘瀽

result1<-gsea@result

gsea@result$Description<-tolower(gsea@result$Description)

p<-gseaplot2(gsea,1,color="red",pvalue_table = T) # 鎸夌涓€涓仛浜岀淮鐮佸浘锛屽苟鏄剧ずp鍊?

ggsave(p,file="gsea_go1.pdf",width =8,height =6)

p<-gseaplot2(gsea,2,color="red",pvalue_table = T) # 鎸夌涓€涓仛浜岀淮鐮佸浘锛屽苟鏄剧ずp鍊?

ggsave(p,file="gsea_go2.pdf",width =8,height =6)

p<-gseaplot2(gsea,3,color="red",pvalue_table = T) # 鎸夌涓€涓仛浜岀淮鐮佸浘锛屽苟鏄剧ずp鍊?

ggsave(p,file="gsea_go3.pdf",width =8,height =6)

p<-gseaplot2(gsea,4,color="red",pvalue_table = T) # 鎸夌涓€涓仛浜岀淮鐮佸浘锛屽苟鏄剧ずp鍊?

ggsave(p,file="gsea_go4.pdf",width =8,height =6)

p<-gseaplot2(gsea,5,color="red",pvalue_table = T) # 鎸夌涓€涓仛浜岀淮鐮佸浘锛屽苟鏄剧ずp鍊?

ggsave(p,file="gsea_go5.pdf",width =8,height =6)

write.table(gsea@result,"m1_go.txt",row.names = F,col.names = T,sep = "\t",quote = F)

write.csv(gsea@result,"m1_go.csv",row.names = F)

gsea1<-gsea@result[gsea@result$enrichmentScore>0,]

gsea1<-gsea1[order(gsea1$p.adjust),]

gsea2<-gsea@result[gsea@result$enrichmentScor<0,]

gsea2<-gsea2[order(gsea2$p.adjust),]

k1<-ggplot(gsea1[1:25,],aes(x=enrichmentScore,y=Description))+

geom_point(aes(size=setSize,color=p.adjust))+

scale_color_gradient(low="#66A61E",high ="#E6AB02")+

labs(color=expression(p.adjust),size="count",x="enrichmentScore",y="")+

theme_bw()

ggsave(k1,file="gsea_go1.pdf",width =12,height =15)

k1<-ggplot(gsea2[1:25,],aes(x=enrichmentScore,y=Description))+

geom_point(aes(size=setSize,color=p.adjust))+

scale_color_gradient(low="#66A61E",high ="#E6AB02")+

labs(color=expression(p.adjust),size="count",x="enrichmentScore",y="")+

theme_bw()

ggsave(k1,file="gsea_go2.pdf",width =8,height =10)

#Figure 7

rm(list = ls())

setwd("D:\\job\\SNOW2021\\3-cad\\9-ppi")

#ppi

ppi<-read.table("string_interactions_short.tsv",header =F,sep = "\t")

ppi<-ppi[,1:2]

#ppi <- unique(as.data.frame(t(apply(ppi, 1, sort))))

library(xlsx)

colnames(ppi)<-c("gene1","gene2")

write.table(ppi,"ppi_string_output.txt",row.names = F,sep = "\t",col.names = T,quote = F)

write.xlsx2(ppi,"ppi_string_output.xls",row.names = F,col.names = T)

gene_freq<-as.data.frame(table(c(ppi$gene1,ppi$gene2)))

gene_freq<-gene_freq[order(gene_freq$Freq,decreasing = T),]

write.table(gene_freq,"gene_freq_output.txt",row.names = F,sep = "\t",col.names = T,quote = F)

write.xlsx2(gene_freq,"gene_freq_output.xls",row.names = F,col.names = T)

write.table(gene_freq$Var1,"ppi_gene_output.txt",row.names = F,sep = "\t",col.names = T,quote = F)

write.xlsx2(gene_freq$Var1,"ppi_gene_output.xls",row.names = F,col.names = T)

m1<-read.csv("ppi_string_output.txt default edge.csv")

m11<-strsplit(m1$shared.name,split='\\ ')

gene2<-c()

for (i in 1:nrow(m1)) {

gene2<-c(gene2,m11[[i]][1],m11[[i]][4])}

gene2<-unique(gene2)

write.table(gene2,"ppi_gene.txt",row.names = F,sep = "\t",col.names = F,quote = F)

m1<-read.csv("ppi_string_output.txt_MCC_top30 default edge.csv")

m11<-strsplit(m1$shared.name,split='\\ ')

gene1<-c()

for (i in 1:nrow(m1)) {

gene1<-c(gene1,m11[[i]][1],m11[[i]][4])}

gene1<-unique(gene1)

write.table(gene1,"gene1.txt",row.names = F,sep = "\t",col.names = F,quote = F)

hub<-gene1

#ceRNA

m2g<-read.csv("gene2mir.csv")

m2g<-m2g[,c(1,3)]

m2g<-m2g[!duplicated(m2g),]

mRNA_freq<-as.data.frame(table(m2g$Target))

miRNA_freq<-as.data.frame(table(m2g$ID))

write.table(m2g,"m-mi.txt",col.names =T,row.names = F,quote = F,sep = "\t")

m2g<-read.csv("gene2mir.csv")

m2g<-m2g[,c(1,3)]

m2g<-m2g[!duplicated(m2g),]

m2l<-read.csv("mir2lnc.csv")

m2l<-m2l[,c(1,3)]

m2l<-m2l[!duplicated(m2l),]

length(unique(m2g$ID))

length(unique(m2l$ID))

length(unique(m2g$Target))

length(unique(m2l$Target))

mi<-intersect(m2g$ID,m2l$ID)

ceRNA<-rbind(m2g,m2l)

ceRNA<-ceRNA[!duplicated(ceRNA),]

mRNA_freq<-as.data.frame(table(m2g$Target))

miRNA_freq<-as.data.frame(table(m2g$ID))

miRNA_freq_1<-as.data.frame(table(m2l$ID))

aa<-intersect(miRNA_freq$Var1,miRNA_freq_1$Var1)

miRNA_freq<-miRNA_freq[which(miRNA_freq$Var1 %in% aa),]

miRNA_freq_1<-miRNA_freq_1[which(miRNA_freq_1$Var1 %in% aa),]

lncRNA_freq<-as.data.frame(table(m2l$Target))

mRNA1<-unique(m2g$Target)

miRNA1<-unique(m2g$ID)

lncRNA<-unique(m2l$Target)

miRNA2<-unique(m2l$ID)

length(unique(c(miRNA1,miRNA2)))

both_miRNA<-intersect(miRNA1,miRNA2)

write.table(ceRNA,"ceRNA.txt",col.names = T,row.names = F,quote = F,sep = "\t")

write.table(miRNA1,"miRNA.txt",col.names = F,row.names = F,quote = F)

write.table(lncRNA,"lncRNA.txt",col.names = F,row.names = F,quote = F)

write.table(mRNA1,"mRNA.txt",col.names = F,row.names = F,quote = F)

write.table(both_miRNA,"both_miRNA.txt",col.names = F,row.names = F,quote = F)

write.table(both_miRNA_1,"both_miRNA_cerna.txt",col.names = T,row.names = F,quote = F,sep = "\t")

m2l<-read.csv("gene2tf.csv")

m2l<-m2l[,c(1,3)]

m2l<-m2l[!duplicated(m2l),]

mRNA_freq1<-as.data.frame(table(m2l$Target))

tf_freq1<-as.data.frame(table(m2l$ID))

write.table(m2l,"m-tf.txt",col.names =T,row.names = F,quote = F,sep = "\t")

options(stringsAsFactors = FALSE) #绂佹chr杞垚factor

library(org.Hs.eg.db)

library(GOSemSim)

library(reshape2)

library(ggplot2)

gene<-as.character(hub)

rt<-data.frame(ENTREZID=mapIds(x = org.Hs.eg.db, keys =gene,keytype = "SYMBOL",column = "ENTREZID"), SYMBOL=gene)

###瀛楃涓茶浆鎹?

rt$ENTREZID <- as.character(rt$ENTREZID)

#2. 璁＄畻鐩镐技鎬?

bp <- godata('org.Hs.eg.db', ont="BP", computeIC = FALSE)

cc <- godata('org.Hs.eg.db', ont="CC", computeIC = FALSE)

mf <- godata('org.Hs.eg.db', ont="MF", computeIC = FALSE)

a<-Reduce(intersect,list(colnames(simbp),colnames(simcc),colnames(simmf)))

rt<-rt[rt$ENTREZID %in% a,]

simbp <- mgeneSim(rt$ENTREZID, semData = bp, measure = "Wang",drop = NULL,combine = "BMA")

simcc <- mgeneSim(rt$ENTREZID, semData = cc, measure = "Wang", drop = NULL,combine = "BMA")

simmf <- mgeneSim(rt$ENTREZID, semData = mf,measure = "Wang",drop = NULL, combine = "BMA")

#璁＄畻鍩哄洜鐨勫嚑浣曞钩鍧囧€?

fsim <- (simmf * simcc * simbp)^(1/3)

#灏嗗熀鍥犵殑鍚嶅瓧鐢盓NTREZID鏀逛负gene symbol

colnames(fsim) = rt$SYMBOL

rownames(fsim) = rt$SYMBOL

#灏嗗熀鍥犺嚜宸卞拰鑷繁鐨勭浉浼煎害璁句负NA

for (i in 1:ncol(fsim)){fsim[i,i] <- NA}

dat <- melt(fsim) #鎶婂鏍煎紡鏁版嵁杞寲鎴愰暱鏍煎紡

dat <- dat[!is.na(dat$value),] #鍒犳帀甯A鐨勮

dat <- dat[,c(1,3)]

head(dat)

#璁＄畻姣忎釜鍩哄洜璺熷叾浠栧熀鍥犵浉浼煎害鐨勫钩鍧囧€?

dat.mean <- aggregate(value~Var1, dat, mean)

m <- dat.mean$value

names(m) <- dat.mean$Var1

#鎸夊钩鍧囧€肩粰鍩哄洜鍚嶆帓搴?

dat$Var1 <- factor(dat$Var1,levels=names(sort(m)))

#缁樺浘

p1<-ggplot(dat,aes(x = Var1, y = value, fill = factor(Var1))) +

geom_boxplot() +scale_colour_gradient(low = "#E6AB02", high = "#66A61E")+

coord_flip() + xlab("") + ylab("") +theme_bw()+theme(legend.position="none")

ggsave(p1,file="friends.pdf",width =8,height =6)

data<-read.csv("D:\\job\\SNOW2021\\3-cad\\2-geo\\adjusted_geo.csv")

rownames(data)<-data$X

data<-data[,-1]

c1<-read.table("D:\\job\\SNOW2021\\3-cad\\7-subgroup\\c1.txt")

c2<-read.table("D:\\job\\SNOW2021\\3-cad\\7-subgroup\\c2.txt")

data<-data[,c(c1$V1,c2$V1)]

data2 <-as.data.frame( t(data[which(rownames(data) %in% gene),]))

data2$cla<-c(rep('c1',83),rep('c2',159))

library(pROC)

for (i in 1:26) {

roc1<- roc(data2$cla,data2[,i],smooth =T,ci=F)

if(roc1$auc>0.7){

aa<-paste0(gene[i],".pdf")

pdf(aa,width = 6,height = 5)

plot(roc1,main=gene[i])

plot(roc1, col="#E6AB02",print.auc=TRUE, add=TRUE, grid=c(0.1, 0.2), print.auc.x=0.45,print.auc.y=0.1)

dev.off()

}else next

}

library(clusterProfiler)

library(org.Hs.eg.db)

library(xlsx)

library(ggplot2)

library(GOplot)

library(stringr)

library(enrichplot)

library(limma)

limma<-read.table("D:\\job\\SNOW2021\\3-cad\\8-enrich\\cluster-limma.txt")

genelist<-EC$genelist

david<-EC$david

limma<-limma[,c(7,1:6)]

colnames(limma)<-colnames(genelist)

david_m<-read.table("all_gene_david.txt",header = T,sep = "\t",quote = "")

david_m<-david_m[which(david_m$PValue<0.05),]

ego_result_ALL<-david_m[which(substr(david_m$Category,1,6) %in% 'GOTERM'),]

ego_result_ALL$Category<-substr(ego_result_ALL$Category,8,9)

ego_result_ALL$ID<-substr(ego_result_ALL$Term,1,10)

ego_result_ALL$Term<-substr(ego_result_ALL$Term,12,nchar(ego_result_ALL$Term))

ego_result_ALL<-ego_result_ALL[,c(1,14,2,6,5)]

colnames(ego_result_ALL)<-colnames(david)

write.table(ego_result_ALL,"deg_go_output.txt",row.names = F,quote = F,sep = "\t")

write.csv(ego_result_ALL,"deg_go_output.csv",row.names=F)

bp<- ego_result_ALL[which(ego_result_ALL$Category %in% "BP"),][1:15,]

cc<- ego_result_ALL[which(ego_result_ALL$Category %in% "CC"),][1:15,]

mf<- ego_result_ALL[which(ego_result_ALL$Category %in% "MF"),][1:15,]

ego_result_ALL<-rbind(bp,cc,mf)

ego_result_ALL<-na.omit(ego_result_ALL)

circ <- circle_dat(ego_result_ALL,limma)

pdf("all_gene_GO.pdf",width = 12,height = 9)

GOBubble(circ, title = '', colour = c("#E6AB02", "#66A61E","#9966FF"), display = 'multiple', labels = 5,table.legend = F)

dev.off()

david_m<-read.table("gene1_david.txt",header = T,sep = "\t",quote = "")

david_m<-david_m[which(david_m$PValue<0.05),]

ego_result_ALL<-david_m[which(substr(david_m$Category,1,6) %in% 'GOTERM'),]

ego_result_ALL$Category<-substr(ego_result_ALL$Category,8,9)

ego_result_ALL$ID<-substr(ego_result_ALL$Term,1,10)

ego_result_ALL$Term<-substr(ego_result_ALL$Term,12,nchar(ego_result_ALL$Term))

ego_result_ALL<-ego_result_ALL[,c(1,14,2,6,5)]

colnames(ego_result_ALL)<-colnames(david)

write.table(ego_result_ALL,"gene1_go_output.txt",row.names = F,quote = F,sep = "\t")

write.csv(ego_result_ALL,"gene1_go_output.csv",row.names=F)

bp<- ego_result_ALL[which(ego_result_ALL$Category %in% "BP"),][1:15,]

cc<- ego_result_ALL[which(ego_result_ALL$Category %in% "CC"),][1:9,]

mf<- ego_result_ALL[which(ego_result_ALL$Category %in% "MF"),][1:11,]

ego_result_ALL<-rbind(bp,cc,mf)

ego_result_ALL<-na.omit(ego_result_ALL)

circ <- circle_dat(ego_result_ALL,limma)

pdf("gene1_GO.pdf",width = 12,height = 9)

GOBubble(circ, title = '', colour = c("#E6AB02", "#66A61E","#9966FF"), display = 'multiple', labels = 5,table.legend = F)

dev.off()

#Figure 8

rm(list=ls())

setwd("D:\\job\\SNOW2021\\3-cad\\10-ssgsea")

data<-read.csv("D:\\job\\SNOW2021\\3-cad\\2-geo\\adjusted_geo.csv")

rownames(data)<-data$X

data<-data[,-1]

c1<-read.table("D:\\job\\SNOW2021\\3-cad\\7-subgroup\\c1.txt")

c2<-read.table("D:\\job\\SNOW2021\\3-cad\\7-subgroup\\c2.txt")

data<-data[,c(c1$V1,c2$V1)]

library(GSVA)

library(ComplexHeatmap)

library(ggplot2)

cell_gene<-read.table("Immune marker genes.txt",sep=",")

cell<-unique(as.character(cell_gene[,2]))

cell_gene_type=list()

for (i in 1:length(cell)){

b=list(as.character(cell_gene[(as.character(cell_gene$V2))==cell[i],1]))

cell_gene_type[i]=(b)

names(cell_gene_type)[i]=cell[i]

}

gsva_es1<-gsva(as.matrix(data),cell_gene_type,method="ssgsea",abs.ranking=F)

library(limma)

group_list <- factor(c(rep('c1',83),rep('c2',159)))

group_list = factor(group_list)

design <- model.matrix(~0+group_list)

rownames(design) = colnames(gsva_es1)

colnames(design) <- levels(group_list)

contrast.matrix<-makeContrasts(paste0(unique(group_list),collapse = "-"),levels = design)

fit <- lmFit(gsva_es1,design)

fit2 <- contrasts.fit(fit, contrast.matrix) ##

fit2 <- eBayes(fit2) ## default no trend !!!

output = topTable(fit2, coef=1, n=Inf)

output1<-output[(output$P.Value<0.01),]

library(xlsx)

write.xlsx(output1,"immu_diff.xls",col.names = T,row.names = T)

cb2<-as.data.frame(t(gsva_es1))

cb2<-cb2[,which(colnames(cb2)%in% rownames(output))]

immu<- rep(colnames(cb2),each=nrow(cb2)) #缁勫埆鍙橀噺

immu <- factor(immu) #缁勫埆鍥犲瓙鍖?

a<-c(rep('c1',83),rep('c2',159))

group <- rep(a,ncol(cb2)) #姣忎釜缁勫埆鐨勪袱涓睘鎬?

group <- factor(group) #灞炴€у洜瀛愬寲

value <- c() #闅忔満璧嬪€?

for (j in 1:ncol(cb2)) { value<-c(value,cb2[,j])}

value<-as.numeric(value)

Data <- data.frame(immu_cell=immu,group=group,value=value) #鐢熸垚鏁版嵁妗?

write.table(Data,"A.txt",sep = "\t",quote = F)

library(ggplot2)

library(corrgram)

library(ggthemes)

library(ggpubr)

p<-ggplot(Data,aes(x=immu_cell,y=value,fill=group))+

geom_boxplot(width=0.7,size=0.3,outlier.color = NA)+

theme_bw()+scale_fill_manual(values = c("#E6AB02", "#66A61E"))+

theme(panel.grid = element_blank())+

stat_compare_means(symnum.args = list(cutpoints = c(0,0.001, 0.01, 0.05, 1),

symbols = c("***", "**", "*", "ns")),label = "p.signif")+

theme(axis.text.x = element_text(angle = 90,hjust = 1))+

theme(legend.position = 'top')+xlab('')+ylab('Infiltration Abundance')+labs(fill='Group')

ggsave(p,file="immu_barplot2.pdf",width = 15,height = 8)

immu<-t(gsva_es1[which(rownames(gsva_es1)%in% rownames(output1)),1:83])

write.table(immu,"C.txt",sep = "\t",quote = F)

library(corrplot)

pdf("immu_relative_c1.pdf",width = 11,height = 8)

corrplot(corr =cor(immu),order="AOE",type="upper",tl.pos="tp",method="number")

corrplot(corr = cor(immu),add=TRUE, type="lower", method="pie",order="AOE", diag=FALSE,tl.pos="n", cl.pos="n")

dev.off()

immu<-t(gsva_es1[which(rownames(gsva_es1)%in% rownames(output1)),84:242])

write.table(immu,"D.txt",sep = "\t",quote = F)

library(corrplot)

pdf("immu_relative_c2.pdf",width = 11,height = 8)

corrplot(corr =cor(immu),order="AOE",type="upper",tl.pos="tp",method="number")

corrplot(corr = cor(immu),add=TRUE, type="lower", method="pie",order="AOE", diag=FALSE,tl.pos="n", cl.pos="n")

dev.off()

group_list <- factor(c(rep('c1',83),rep('c2',159)))

bb<-read.table("D:\\job\\SNOW2021\\3-cad\\9-ppi\\gene1.txt")

bb<-bb$V1

data1<-data[bb,]

library(ggplot2)

library(dplyr)

library(Hmisc)

library("Rmisc")

library("plyr")

immu1<-cbind(t(gsva_es1[,]),t(data1[,]))

cor<-data.frame()

for (mm in 29:58) {

cor1<-data.frame(0,0,0,0)

for (i in 1:28) {

c<-rcorr(immu1[,i],immu1[,mm])

cor1[i,1]<-c$r[2]

cor1[i,2]<-c$P[2]

cor1[i,3]<-colnames(immu1)[i]

cor1[i,4]<-colnames(immu1)[mm]

}

cor<-rbind(cor,cor1)

}

colnames(cor)<-c("correlation","Pvalue","immu_cell","gene")

cor4<-cor[cor$Pvalue<0.05,]

cor4<-na.omit(cor4)

cor4<-cor4[order(cor4$correlation,decreasing = F),]

write.table(cor4,"B.txt",sep = "\t",quote = F)

y=factor(cor4$gene)

x=factor(cor4$immu_cell)

a<-(-log10(cor4$Pvalue))

p<-ggplot(cor4,aes(x,y))+ geom_point(aes(size=a,color=correlation))+

scale_colour_gradient(high="#E6AB02",low="#66A61E")+

theme(plot.title = element_text(hjust = 0.5))+

theme_classic()+labs(size="-log10Pvalue",x="",y="")+

theme(axis.text.x = element_text(angle = 90, hjust = 1, vjust = 0.5))

ggsave(p,file="immu-28.pdf",width =8,height =6)

immu1<-cbind(t(gsva_es1[,1:83]),t(data1[,1:83]))

cor<-data.frame()

for (mm in 29:58) {

cor1<-data.frame(0,0,0,0)

for (i in 1:28) {

c<-rcorr(immu1[,i],immu1[,mm])

cor1[i,1]<-c$r[2]

cor1[i,2]<-c$P[2]

cor1[i,3]<-colnames(immu1)[i]

cor1[i,4]<-colnames(immu1)[mm]

}

cor<-rbind(cor,cor1)

}

colnames(cor)<-c("correlation","Pvalue","immu_cell","gene")

cor4<-cor[cor$Pvalue<0.05,]

cor4<-na.omit(cor4)

cor4<-cor4[order(cor4$correlation,decreasing = F),]

write.table(cor4,"E.txt",sep = "\t",quote = F)

y=factor(cor4$gene)

x=factor(cor4$immu_cell)

a<-(-log10(cor4$Pvalue))

p<-ggplot(cor4,aes(x,y))+ geom_point(aes(size=a,color=correlation))+

scale_colour_gradient(high="#E6AB02",low="#66A61E")+

theme(plot.title = element_text(hjust = 0.5))+

theme_classic()+labs(size="-log10Pvalue",x="",y="")+

theme(axis.text.x = element_text(angle = 90, hjust = 1, vjust = 0.5))

ggsave(p,file="immu-28-c1.pdf",width =8,height =6)

immu1<-cbind(t(gsva_es1[,84:242]),t(data1[,84:242]))

cor<-data.frame()

for (mm in 29:58) {

cor1<-data.frame(0,0,0,0)

for (i in 1:28) {

c<-rcorr(immu1[,i],immu1[,mm])

cor1[i,1]<-c$r[2]

cor1[i,2]<-c$P[2]

cor1[i,3]<-colnames(immu1)[i]

cor1[i,4]<-colnames(immu1)[mm]

}

cor<-rbind(cor,cor1)

}

colnames(cor)<-c("correlation","Pvalue","immu_cell","gene")

cor4<-cor[cor$Pvalue<0.05,]

cor4<-na.omit(cor4)

cor4<-cor4[order(cor4$correlation,decreasing = F),]

write.table(cor4,"F.txt",sep = "\t",quote = F)

y=factor(cor4$gene)

x=factor(cor4$immu_cell)

a<-(-log10(cor4$Pvalue))

p<-ggplot(cor4,aes(x,y))+ geom_point(aes(size=a,color=correlation))+

scale_colour_gradient(high="#E6AB02",low="#66A61E")+

theme(plot.title = element_text(hjust = 0.5))+

theme_classic()+labs(size="-log10Pvalue",x="",y="")+

theme(axis.text.x = element_text(angle = 90, hjust = 1, vjust = 0.5))

ggsave(p,file="immu-28-c2.pdf",width =8,height =6)

#Figure 9

rm(list = ls())

setwd("D:\\job\\SNOW2021\\3-cad\\11-cibersort")

data<-read.csv("D:\\job\\SNOW2021\\3-cad\\2-geo\\adjusted_geo.csv")

rownames(data)<-data$X

data<-data[,-1]

c1<-read.table("D:\\job\\SNOW2021\\3-cad\\7-subgroup\\c1.txt")

c2<-read.table("D:\\job\\SNOW2021\\3-cad\\7-subgroup\\c2.txt")

data<-data[,c(c1$V1,c2$V1)]

library(limma)

library(FactoMineR)

library(factoextra)

library(ggplot2)

library(corrgram)

library(ggthemes)

library(ggpubr)

table_cell<-read.table('LM22_input.txt',fill=T,header=T,sep='\t',row.names=1,check.names=F)

#data1

##############################################################

exp1<-data

exp1<-exp1[intersect(rownames(exp1),rownames(table_cell)),]

table_cell1<-table_cell[intersect(rownames(exp1),rownames(table_cell)),]

source('cibersort.R')

result <- CIBERSORT(table_cell1,exp1, perm = 500, QN = T)

write.table(as.data.frame(result)[,1:22],"ciber_res1.txt",row.names = T,sep = "\t")

cb1=as.data.frame(result)[,1:22]

group_list <- factor(c(rep('c1',83),rep('c2',159)))

group_list = factor(group_list)

design <- model.matrix(~0+group_list)

rownames(design) = colnames(t(cb1))

colnames(design) <- levels(group_list)

contrast.matrix<-makeContrasts(paste0(unique(group_list),collapse = "-"),levels = design)

fit <- lmFit(t(cb1),design)

fit2 <- contrasts.fit(fit, contrast.matrix) ##

fit2 <- eBayes(fit2) ## default no trend !!!

output = topTable(fit2, coef=1, n=Inf)

write.table(output,"B",row.names = T,sep = "\t")

output1<-output[(output$P.Value<0.05),]

cb11<-t(cb1)

deg_h<-cb11[which(rownames(cb11) %in% rownames(output)),]

cb11<-as.data.frame(t(deg_h))

patient<- rep(rownames(cb11),each=ncol(cb11)) #缁勫埆鍙橀噺

group <-rep(colnames(cb11),nrow(cb11))

value <- c() #闅忔満璧嬪€?

for (j in 1:nrow(cb11)) {value<-c(value,cb11[j,])}

value<-abs(as.numeric(value))

Data <- data.frame(patient_id=patient,group=group,value=value) #鐢熸垚鏁版嵁妗?

colour <- c("#DC143C","#0000FF","#20B2AA","#FFA500","#9370DB","#98FB98","#F08080","#1E90FF","#7CFC00","#FFFF00","#808000","#FF00FF","#FA8072","#7B68EE","#9400D3","#800080","#A0522D","#D2B48C","#D2691E","#87CEEB","#40E0D0","#5F9EA0","#FF1493","#0000CD","#008B8B","#FFE4B5","#8A2BE2","#228B22","#E9967A","#4682B4","#32CD32","#F0E68C","#FFFFE0","#EE82EE","#FF6347","#6A5ACD","#9932CC","#8B008B","#8B4513","#DEB887")

p<-ggplot(Data,aes(patient_id,value,fill=group))+ theme(legend.position = "bottom")+

geom_bar(stat="identity",position="fill")+ylab("")+xlab("")+

ggtitle("")+ theme_bw()+theme(axis.text.x = element_text(angle = 90, hjust = 0.5, vjust = 0.5))+

guides(fill=guide_legend(title=NULL)) +scale_fill_manual(values =colour )

ggsave(p,file="immu1.pdf",width = 15,height =8)

cb2<-as.data.frame(cb1)

immu<- rep(colnames(cb2),each=nrow(cb2)) #缁勫埆鍙橀噺

immu <- factor(immu) #缁勫埆鍥犲瓙鍖?

a<-c(rep('c1',83),rep('c2',159))

group <- rep(a,ncol(cb2)) #姣忎釜缁勫埆鐨勪袱涓睘鎬?

group <- factor(group) #灞炴€у洜瀛愬寲

value <- c() #闅忔満璧嬪€?

for (j in 1:ncol(cb2)) { value<-c(value,cb2[,j])}

value<-as.numeric(value)

Data <- data.frame(immu_cell=immu,group=group,value=value) #鐢熸垚鏁版嵁妗?

library(ggplot2)

library(corrgram)

library(ggthemes)

library(ggpubr)

p<-ggplot(Data,aes(x=immu_cell,y=value,fill=group))+

geom_boxplot(width=0.7,size=0.3,outlier.color = NA)+

theme_bw()+scale_fill_manual(values = c("#E6AB02", "#66A61E"))+

theme(panel.grid = element_blank())+

stat_compare_means(symnum.args = list(cutpoints = c(0,0.001, 0.01, 0.05, 1),

symbols = c("***", "**", "*", "ns")),label = "p.signif")+

theme(axis.text.x = element_text(angle = 90,hjust = 1))+

theme(legend.position = 'top')+xlab('')+ylab('Infiltration Abundance')+labs(fill='Group')

ggsave(p,file="immu_barplot2.pdf",width = 15,height = 8)

gsva_es1<-t(cb1)

immu<-t(gsva_es1[which(rownames(gsva_es1)%in% rownames(output1)),1:83])

library(corrplot)

pdf("immu_relative_c1.pdf",width = 11,height = 8)

corrplot(corr =cor(immu),order="AOE",type="upper",tl.pos="tp",method="number")

corrplot(corr = cor(immu),add=TRUE, type="lower", method="pie",order="AOE", diag=FALSE,tl.pos="n", cl.pos="n")

dev.off()

immu<-t(gsva_es1[which(rownames(gsva_es1)%in% rownames(output1)),84:242])

library(corrplot)

pdf("immu_relative_c2.pdf",width = 11,height = 8)

corrplot(corr =cor(immu),order="AOE",type="upper",tl.pos="tp",method="number")

corrplot(corr = cor(immu),add=TRUE, type="lower", method="pie",order="AOE", diag=FALSE,tl.pos="n", cl.pos="n")

dev.off()

bb<-read.table("D:\\job\\SNOW2021\\3-cad\\9-ppi\\gene1.txt")

bb<-bb$V1

data1<-data[bb,]

library(ggplot2)

library(dplyr)

library(Hmisc)

library("Rmisc")

library("plyr")

immu1<-cbind(cb1,t(data1))

cor<-data.frame()

for (mm in 23:51) {

cor1<-data.frame(0,0,0,0)

for (i in 1:22) {

c<-rcorr(immu1[,i],immu1[,mm])

cor1[i,1]<-c$r[2]

cor1[i,2]<-c$P[2]

cor1[i,3]<-colnames(immu1)[i]

cor1[i,4]<-colnames(immu1)[mm]

}

cor<-rbind(cor,cor1)

}

colnames(cor)<-c("correlation","Pvalue","immu_cell","gene")

cor4<-cor[cor$Pvalue<0.05,]

cor4<-na.omit(cor4)

cor4<-cor4[order(cor4$correlation,decreasing = F),]

y=factor(cor4$gene)

x=factor(cor4$immu_cell)

a<-(-log10(cor4$Pvalue))

p<-ggplot(cor4,aes(x,y))+ geom_point(aes(size=a,color=correlation))+

scale_colour_gradient(high="#E6AB02",low="#66A61E")+

theme(plot.title = element_text(hjust = 0.5))+

theme_classic()+labs(size="-log10Pvalue",x="",y="")+

theme(axis.text.x = element_text(angle = 90, hjust = 1, vjust = 0.5))

ggsave(p,file="immu-22.pdf",width =8,height =6)

immu1<-cbind(cb1[1:83,],t(data1[,1:83]))

cor<-data.frame()

for (mm in 23:51) {

cor1<-data.frame(0,0,0,0)

for (i in 1:22) {

c<-rcorr(immu1[,i],immu1[,mm])

cor1[i,1]<-c$r[2]

cor1[i,2]<-c$P[2]

cor1[i,3]<-colnames(immu1)[i]

cor1[i,4]<-colnames(immu1)[mm]

}

cor<-rbind(cor,cor1)

}

colnames(cor)<-c("correlation","Pvalue","immu_cell","gene")

cor4<-cor[cor$Pvalue<0.05,]

cor4<-na.omit(cor4)

cor4<-cor4[order(cor4$correlation,decreasing = F),]

write.table(cor4,"C.txt",row.names = T,sep = "\t")

y=factor(cor4$gene)

x=factor(cor4$immu_cell)

a<-(-log10(cor4$Pvalue))

p<-ggplot(cor4,aes(x,y))+ geom_point(aes(size=a,color=correlation))+

scale_colour_gradient(high="#E6AB02",low="#66A61E")+

theme(plot.title = element_text(hjust = 0.5))+

theme_classic()+labs(size="-log10Pvalue",x="",y="")+

theme(axis.text.x = element_text(angle = 90, hjust = 1, vjust = 0.5))

ggsave(p,file="immu-22_c1.pdf",width =8,height =6)

immu1<-cbind(cb1[84:242,],t(data1[,84:242]))

cor<-data.frame()

for (mm in 23:51) {

cor1<-data.frame(0,0,0,0)

for (i in 1:22) {

c<-rcorr(immu1[,i],immu1[,mm])

cor1[i,1]<-c$r[2]

cor1[i,2]<-c$P[2]

cor1[i,3]<-colnames(immu1)[i]

cor1[i,4]<-colnames(immu1)[mm]

}

cor<-rbind(cor,cor1)

}

colnames(cor)<-c("correlation","Pvalue","immu_cell","gene")

cor4<-cor[cor$Pvalue<0.05,]

cor4<-na.omit(cor4)

cor4<-cor4[order(cor4$correlation,decreasing = F),]

write.table(cor4,"D.txt",row.names = T,sep = "\t")

y=factor(cor4$gene)

x=factor(cor4$immu_cell)

a<-(-log10(cor4$Pvalue))

p<-ggplot(cor4,aes(x,y))+ geom_point(aes(size=a,color=correlation))+

scale_colour_gradient(high="#E6AB02",low="#66A61E")+

theme(plot.title = element_text(hjust = 0.5))+

theme_classic()+labs(size="-log10Pvalue",x="",y="")+

theme(axis.text.x = element_text(angle = 90, hjust = 1, vjust = 0.5))

ggsave(p,file="immu-22_c2.pdf",width =8,height =6)
